# Adenosine deaminase modulates metabolic remodeling and orchestrates joint destruction in rheumatoid arthritis

**DOI:** 10.1038/s41598-021-94607-5

**Published:** 2021-07-23

**Authors:** Sai Krishna Srimadh Bhagavatham, Prakash Khanchandani, Vishnu Kannan, Damodaram Potikuri, Divya Sridharan, Sujith Kumar Pulukool, Ashwin Ashok Naik, Rajesh Babu Dandamudi, Sai Mangala Divi, Ashish Pargaonkar, Rahul Ray, Saibharath Simha Reddy Santha, Polani B. Seshagiri, K. Narasimhan, Narsimulu Gumdal, Venketesh Sivaramakrishnan

**Affiliations:** 1 Disease Biology Lab, Department of Biosciences, Sri Sathya Sai Institute of Higher Learning, Puttaparthi, 515134 India; 2 Department of Orthopedics, Sri Sathya Sai Institute of Higher Medical Sciences, PG, Puttaparthi, 515134 India; 3 Sri Sathya Sai General Hospital, Puttaparthi, 515134 India; 4 Molecular Reproduction and Developmental Genetics, Indian Institute of Science, Bengaluru, 560012 India; 5 Department of Chemistry, Sri Sathya Sai Institute of Higher Learning, Prasanthi Nilayam, India; 6 Department of Biochemistry, Sri Sathya Sai Institute of Higher Medical Sciences, PG, Puttaparthi, 515134 India; 7 Agilent Technologies India Pvt Ltd, Bengaluru, 560048 India; 8 Present Address: Department of Botany/Biotechnology, CMS College, Kottayam, 686001 India; 9 Present Address: Phenomenex India, Hyderabad, Telangana 500084 India

**Keywords:** Biochemistry, Cell biology, Computational biology and bioinformatics, Biomarkers, Diseases, Rheumatic diseases, Rheumatoid arthritis

## Abstract

Rheumatoid Arthritis (RA) is a chronic autoimmune disease associated with inflammation and joint remodeling. Adenosine deaminase (ADA), a risk factor in RA, degrades adenosine, an anti-inflammatory molecule, resulting in an inflammatory bias. We present an integrative analysis of clinical data, cytokines, serum metabolomics in RA patients and mechanistic studies on ADA-mediated effects on in vitro cell culture models. ADA activity differentiated patients into low and high ADA sets. The levels of the cytokines TNFα, IFNγ, IL-10, TGFβ and sRANKL were elevated in RA and more pronounced in high ADA sets. Serum metabolomic analysis shows altered metabolic pathways in RA which were distinct between low and high ADA sets. Comparative analysis with previous studies shows similar pathways are modulated by DMARDs and biologics. Random forest analysis distinguished RA from control by methyl-histidine and hydroxyisocaproic acid, while hexose-phosphate and fructose-6-phosphate distinguished high ADA from low ADA. The deregulated metabolic pathways of High ADA datasets significantly overlapped with high ADA expressing PBMCs GEO transcriptomics dataset. ADA induced the death of chondrocytes, synoviocyte proliferation, both inflammation in macrophages and their differentiation into osteoclasts and impaired differentiation of mesenchymal stem cells to osteoblasts and mineralization. PBMCs expressing elevated ADA had increased expression of cytokines and P2 receptors compared to synovial macrophages which has low expression of ADA. Our data demonstrates increased cytokine levels and distinct metabolic signatures of RA based on the ADA activity, suggests an important role for ADA in the pathophysiology of RA joints and as a potential marker and therapeutic target in RA patients.

## Introduction

RA is a chronic systemic autoimmune disease with joint inflammation, erosions and deformities^1,2^. RA has a worldwide prevalence of 1%, with a female to male ratio of 3:1 ratio^3,4^. Uncontrolled RA with active inflammation affects joint remodeling leading to degradation of cartilage, proliferation of synoviocytes, reduced bone mineralization and enhanced differentiation of macrophages into osteoclasts. Controlling RA disease involves frequent follow up of the disease activity which help to manage the disease. Despite the availability of many tests for diagnosis and prognosis of RA a gold standard test to evaluate the disease activity of RA is lacking. Current methods rely on Disease Activity Score (DAS) of the tender and swollen joints (in a total of 28-joints both small and large). This may not provide a complete measure for treating the patients as taking the counts is subjective, complex and prone to errors. ESR and CRP can act as better predictors of RA disease progression^5^. But they are useful for understanding the short-term inflammatory changes in the patients. ADA, an enzyme of purine metabolism which plays an important role in cell mediated immunity, invokes inflammation and is associated with many inflammatory and infectious diseases^6–8^. ADA reflects the cell mediated immunity and also involved in maturation of lymphocytes and their function, formation of macrophages from monocytes^9^. RA is associated with increased levels of ADA in the serum and synovial fluid of joints^10,11^. ADA has been suggested as a marker to evaluate disease activity in RA by previous studies^12,13^. Mechanistically, ADA is thought to induce inflammation by modulating nucleotide signaling^14^.

In mammals, nucleotide signaling activates the P2 receptors—P2X and P2Y and is countered by adenosine signaling ensuring homeostasis^15^. Adenosine triphosphate (ATP) is degraded by ectonucleotidases CD39 and CD73 by dephosphorylation to ADP, AMP and to adenosine extracellularly^14^. Extracellular adenosine is scavenged by ADA which shifts the balance towards P2 receptor signaling thus eliciting a pro-inflammatory response^16^. Nucleotide signalling which includes the P2 receptors and adenosine signaling play major roles in joint remodeling, by modulating the function of chondrocytes, synoviocytes, osteoblasts, osteoclasts and immune cells^17,18^. Osteoclastogenesis mediated by the receptor activator of nuclear factor kappa-B ligand (RANKL) is dependent on P2 receptors^19^. Using P2 receptor antagonists improved disease pathology in collagen induced arthritis of mice^14^. Methotrexate (MTX), a disease-modifying anti-rheumatic drug (DMARD) widely used in the treatment of RA, is reported to increase extracellular levels of adenosine^20^.

The inflammatory response in RA is associated with the production of cytokines (TNFα, IL-6, IL-17, IL-20) by innate and adaptive immunity, osteoblasts, synoviocytes, and chondrocytes^21,22^. The cytokines are linked to the degradation of cartilage, proliferation of synoviocytes, and osteoclastogenesis which contribute to pathophysiology of RA^23^. ADA may exacerbate the pathogenesis of RA by enhancing the inflammatory response leading to secretion of cytokines which might drive disease outcomes in RA.

Omics studies have contributed significantly to our understanding of the outcomes associated with RA^24^. Genomics studies have led to the identification of several genes as markers, phenotype prediction of disease severity and drug response^25^. Proteomics studies have shown distinct cytokine profiles and enzyme levels^26,27^ while metabolomic studies have led to the elucidation of metabolic profile in blood and synovial fluid from RA^28,29^. The Omics studies on RA patients can help elucidate underlying pathophysiological mechanisms and elucidate potential biomarkers. However, metabolites being the cumulative endpoint of the changes in genomics, epigenomics, transcriptomics and proteomic profiles, is potentially a better tool for predicting the molecular signatures of the disease process in RA.

In view of the above, we hypothesize that increased activity of ADA in RA patients drives joint remodeling leading to bone resorption through purinergic and adenosine signaling and altered metabolism. In the current study, we have used a systems biology approach combining clinical parameters, cytokine analysis, and metabolomics of serum from RA patients along with mechanistic studies on macrophage inflammation and differentiation into osteoclasts, chondrocyte cell death, synoviocyte proliferation, and MSCs differentiation to osteoblast and mineralization. Further, we aim to correlate these data with ADA activity to elucidate its possible contribution to joint remodelling and pathophysiology of disease.

## Results

### ADA activity and cytokine profiles are altered in RA patients compared to healthy controls

RA patients recruited to our study (the demographic details were given in the Table 1) were screened for the clinical parameters and also the cytokine profiles by ELISA.

**Table 1:** Demographic details of RA patients.

Total no. of RA patients recruited	58
Total no. of women in %	82.14
Mean age of women in years	45.52 ± 10.19
Mean age of men in years	45.80 ± 13.25
General mean age in years	45.57 ± 10.52
Rheumatoid factor positive in %	92.86

An increase in erythrocyte sedimentation rate (ESR), rheumatoid factor (RF), and ACPAs was observed in the prospective cohort of RA patients. Haematological parameters of RA patients showed an increase in platelets, and a decrease in haematocrit (HCT)/packed cell volume (PCV), and haemoglobin levels, mean corpuscular volume (MCV) at lowest of normal range; WBC, neutrophils count at highest of normal range, and RBC, and lymphocytes were slightly higher (Table 2). The covariates such as age and gender were chosen through correlation analysis. The analysis is expressed as value of covariates (confidence interval (CI) and the range that represents the significance) with odds of covariates as 0.611 (95% CI − 2.004–3.386) for gender, and 0.858 (95% CI − 0.109–0.091) for age. The general linear model was used and univariate analysis of covariates was done to adjust the impact of covariates on ADA activity of RA cohort. Our analysis shows that the age and gender did not impact the clinical parameters of RA cohort.

**Table 2:** Altered levels of clinical parameters in RA patients.

S. no	Clinical parameter	Normal range	Mean	SD	Median	Odds ratio
1	ESR	9–20 mm/h	37.96	24.59	29.50	9.84
2	RA factor latex	0–14 IU/mL	75.12	92.02	26.80	68.20
3	ACPAs	≤ 25 IU/L	402.82	146.01	144.43	43.15
4	WBC	4.0–10.0 × 10^3^/µL	9.59	2.55	9.03	2.81
5	RBC	3.50–5.50 × 10^6^/µL	4.32	0.45	4.22	1.74
6	Neutrophils	2.0–7.0 × 10^3^/µL	6.12	2.07	5.91	13.05
7	Lymphocytes	0.8–4.0 × 10^3^/µL	2.76	1.08	2.63	5.82
8	Platelets	100–300 × 10^3^/µL	328.90	98.68	324.00	46.20
9	HB	12–15 gm/dL	12.03	2.16	11.70	4.49
10	HCT	37.0–54.0%	35.73	5.37	34.60	18.56
11	MCV	80.0–100.0 fL	82.53	7.30	84.80	11.00

S. no.	Erosion scores	Observed range	Mean	SD	Median	
12	Erosion scores (low ADA group)	39–115 (n = 5)	65.2	34.845	69	
13	Erosion scores (high ADA group)	25–273 (n = 13)	98.4	71.386	79	

**Figure 1:**
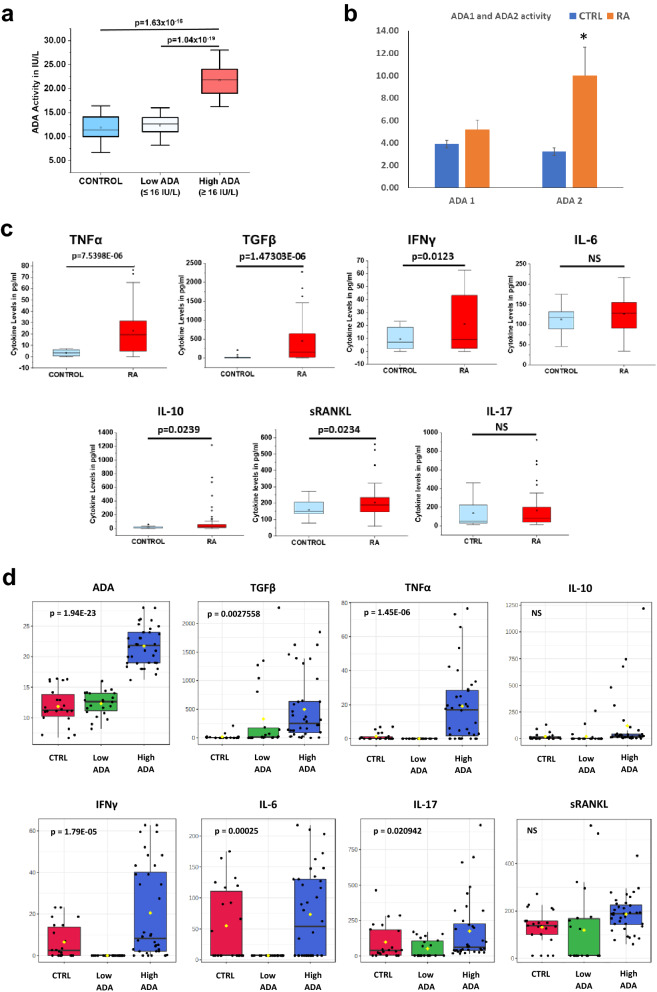
Showing ADA Activity and Cytokine levels in Rheumatoid arthritis patients. (**a**) ADA activity in RA patients representing group with Low ADA and High ADA when compared to healthy controls. (**b**) ADA activity of two isoenzymes ADA 1 and ADA 2 (*representing a p value of ≤ 0.05) (**c**) Levels of Cytokines in the serum of RA patients. (**d**) One-way ANOVA analysis of Cytokines and ADA levels in healthy controls, Low ADA and High ADA RA patients. (p-value of ≤ 0.05 is considered significant). All the plots were made using Origin 2020.

The serum of RA patients had increased ADA activity when compared to healthy controls. We found that RA patients fall into two groups of cohorts based on ADA activity (Fig. 1a). A patient cohort with ADA activity ≤ 16 U/L similar to controls and a second cohort with significantly higher (p < 1.63398E−16) ADA activity > 16 U/L. Further, we also estimated the activity of ADA isoenzymes ADA 1 (intracellular) and ADA 2 (extracellular) in RA patients compared to healthy controls. We observed an increase in ADA 2 activity than ADA 1 activity in the serum samples of RA patients (Fig. 1b).

Total erosion scores were obtained for RA patients by SHARP scoring^30^ (based on X-rays of foot and hand); we observed that many of the RA patients with high ADA levels showed erosion scores ranging from 50 to 160 (Supplementary Fig. S1A).

Significantly elevated levels of TNFα (p = 7.5398E−06), IFNγ (p = 0.0123), IL-10 (p = 0.0239), TGFβ (p = 1.47303E−06), and sRANKL (p = 0.0234) were observed in RA patients compared to healthy controls (Fig. 1c). No significant difference was observed in the level of IL-6 in RA patients compared to controls. RA patients were further divided into two subgroups based on ADA activity i.e., High ADA (> 16 U/L) and Low ADA (≤ 16 U/L). A t-test analysis showed only TNFα and TGFβ to be significant in high ADA groups while the levels were not significant for any cytokines in low ADA groups (Supplementary Fig. S1B). Hence, one-way Analysis of Variance (ANOVA) was used to explore the profile of different cytokines such as TNFα, TGFβ, IFNγ, IL-6, IL-10, IL-17, and sRANKL with ADA levels in RA patients. ANOVA was done followed by a post-hoc analysis (Fisher LSD) to identify potentially significant cytokines that can discriminate between healthy controls, Low ADA and High ADA RA patients. One-way ANOVA showed significant changes in the levels of TNFα, TGFβ, IFNγ, IL-6 and IL-17 (Fig. 1d). However, no change was observed in case of sRANKL and IL10.

### Serum metabolomic analysis of RA patients showed significant changes in metabolic profile compared to healthy controls

Targeted metabolic profiling of serum from healthy controls and RA patients was carried out using Multiple Reaction Monitoring (MRM). We examined qualitative levels of about 200 metabolites through LC MS/MS and could detect 124 metabolites in both positive and negative ionization across 43 samples (n = 16 healthy controls and n = 27 RA patients) (Supplementary Fig. S2). Supplementary Figs. S3 and S4 describe the quality control measures used to ascertain the robustness of the data. Overall, the median coefficient of variance (CV) across 124 metabolites detected was 18.31 (range 9.66–27.57).

**Figure 2:**
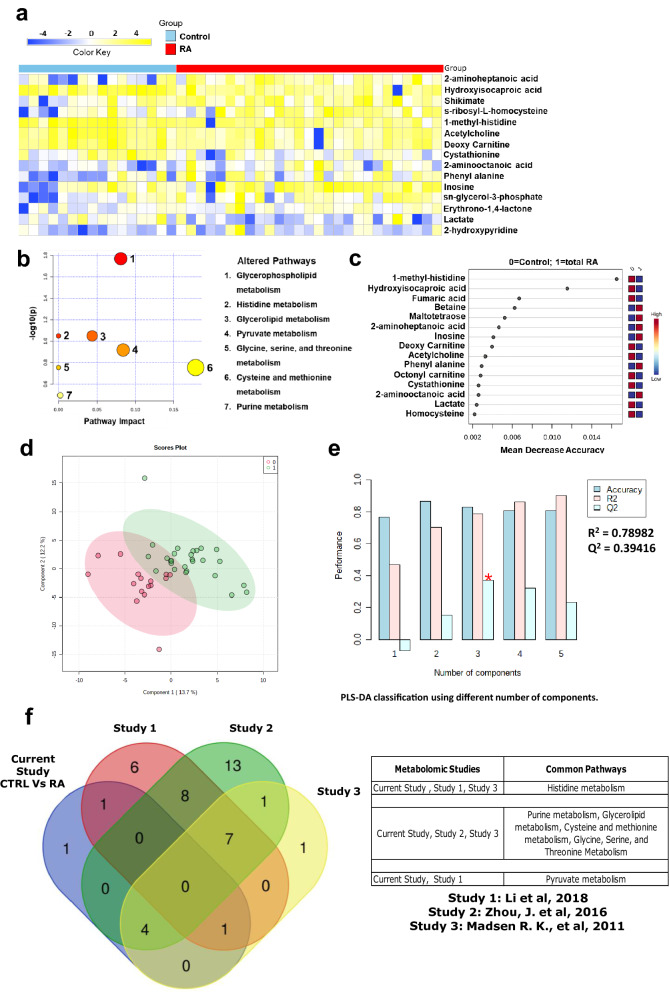
Targeted metabolomics data showing altered metabolic signatures in RA patients compared to healthy controls. (**a**) Heat map showing the relative levels of 15 significantly altered metabolites at FDR 0.25 with shades of yellow and blue representing the elevated and reduced metabolite levels, respectively (heatmaps were made in Microsoft Excel). (**b**) Pathway analysis showing altered metabolic pathways in RA patients compared to healthy controls. Size of the circle represents the impact of each pathway and with color representing level of significance (highest in red to lowest in yellow). (**c**) Ranking of significant metabolites based on random forest analysis for their ability to stratify RA patients from healthy controls (ranked by the mean decrease in classication accuracy when they are permuted). (**d**) PLS-DA clustering given by score plot (0 = Control, 1 = RA) and (**e**) cross validation of PLS-DA of RA patients with healthy controls (red star indicates the best classifier based on accuracy, R^2^, and Q^2^ values). (Figures b, c, d, and e were generated using the web-based tool Metaboanalyst https://www.metaboanalyst.ca/). (**f**) Overlapping pathways between our serum metabolomics data of RA patients with three independent published serum metabolomics datasets of RA patients. (Venn Diagram was created using an online tool http://bioinformatics.psb.ugent.be/webtools/Venn/).

Fifteen metabolites were significantly altered at a False Discovery Rate (FDR) of less than 0.25 between RA and healthy controls (Supplementary Table S1). These included nine metabolites that were elevated and six that were reduced in RA samples compared to controls (Fig. 2a). 2-aminoheptanoic acid, and 2-aminooctanoic acid were the most elevated metabolites in RA, while hydroxyisocaproic acid, 1-methyl histidine, and lactate were more prominent in control samples. Consistent with previous reports describing elevated expression of ADA in RA, inosine, a product of ADA activity, was also elevated in RA samples. Pathway mapping of the differential metabolites revealed glycerophospholipid, histidine, and glycerolipid metabolism as top altered pathways (Fig. 2b). Furthermore, a random forest (RF) analysis with 15 predictors and 5000 trees revealed 1-methyl-histidine and hydroxyisocaproic acid (HICA) as significant metabolites that could accurately separate RA patients from healthy controls (Fig. 2c). We performed an unsupervised principal component analysis (PCA) to understand the clustering/distribution of healthy controls and RA patients. We observed less separation between two groups. However, when PLS-DA was done, to know the variance between healthy controls and RA patients, results showed good separation (Fig. 2d) or clustering between the two groups and cross validation (Fig. 2e) resulting in good model parameters (R^2^ = 0.78982, Q^2^ = 0.39416). Permutation test used for validating the PLS-DA model showed that the separation was significant with the p = 0.016 at 1000 permutations. PLS-DA highlighted inosine, octonyl carnitine, and s-ribosyl-l-homocysteine as significant metabolites.

Comparative analysis of serum metabolomics studies of our RA patient cohort was carried out with three published independent serum metabolomics datasets. Results showed significant overlap of pathways that are significantly deregulated (Fig. 2f). We observed that the histidine metabolism from our patient cohort overlapped with the RA serum metabolomics by Li et al., and Madsen et al. Four metabolic pathways purine metabolism, glycerolipid metabolism, cysteine and methionine metabolism, glycine, serine, and threonine metabolism in our patient cohort overlapped with studies by Zhou et al., and Madsen et al. Pyruvate metabolism was found to be overlapping between our study and published dataset by Li et al. (Fig. 2f).

**Figure 3:**
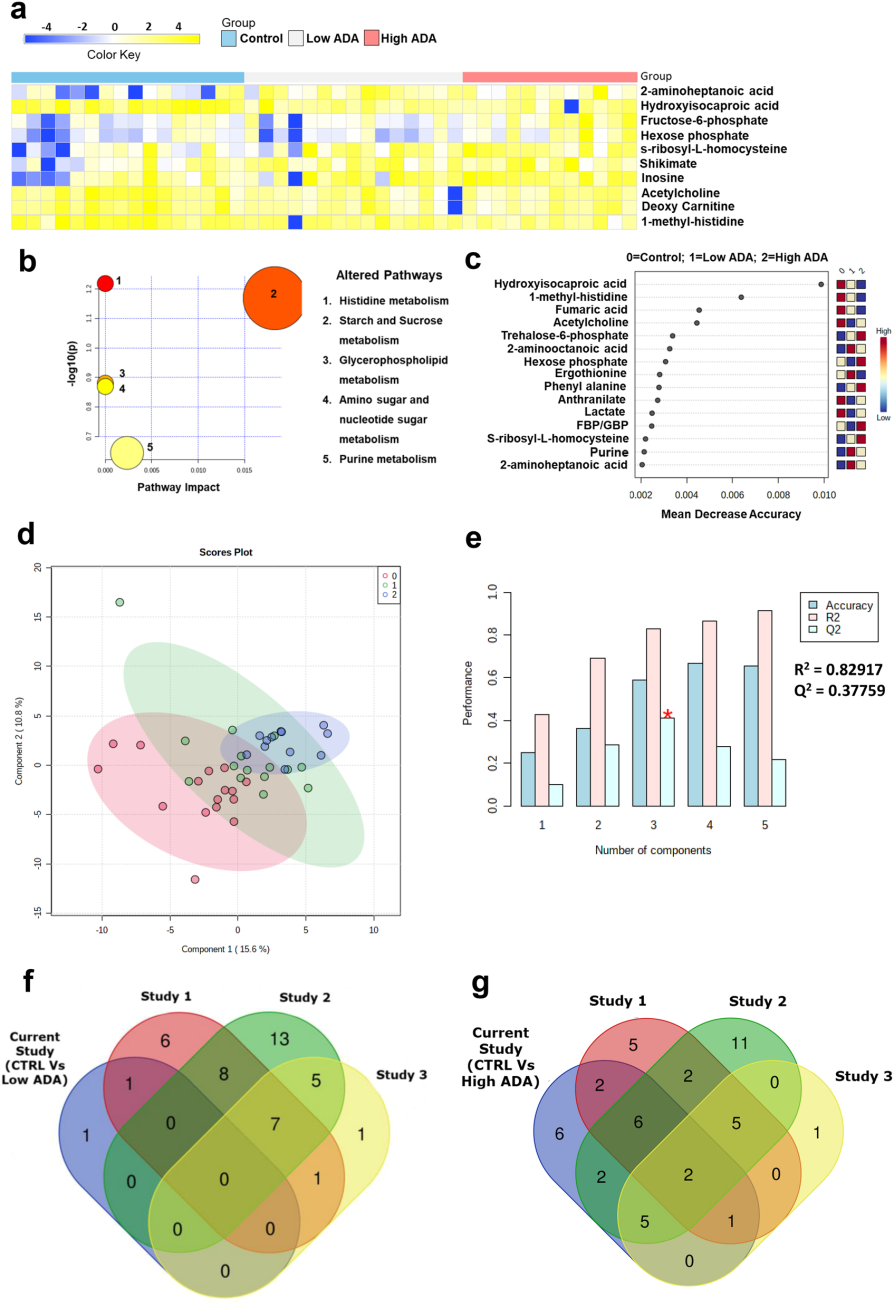
ANOVA analysis comparing metabolic profiles in RA patients with low and high ADA levels and healthy controls. (**a**) Heat map showing the relative levels of 10 significant differential metabolites comparing RA patients with low ADA, high ADA, and healthy controls at FDR 0.25. Shades of yellow and blue represent elevated and reduced levels of metabolites, respectively (heatmaps were made in Microsoft Excel). (**b**) Pathway analysis showing metabolic pathways enriched by the metabolic signature distinguishing RA patients with low ADA, and high ADA, and healthy controls. The size of the circle describes the impact of each pathway in delineating the three groups. The color of the circles represent level of significance (highest in red to lowest in yellow). (**c**) Ranking of significant metabolites based on random forest analysis for their ability to stratify the three groups (ranked by the mean decrease in classication accuracy when they are permuted). (**d**) PLS-DA clustering given by score plot (0 = Control, 1 = Low ADA, 2 = High ADA) and (**e**) cross validation of PLS-DA of RA patients with healthy controls and RA patients with Low and High ADA levels (red star indicates the best classifier based on accuracy, R^2^, and Q^2^ values). (**b**–**d**, and e were generated using the web-based tool Metaboanalyst https://www.metaboanalyst.ca/) (**f**) Overlapping pathways between our serum metabolomics data of Low ADA group and (**g**) of High ADA group with three independent published serum metabolomics datasets of RA patients. (Venn Diagrams were created using an online tool http://bioinformatics.psb.ugent.be/webtools/Venn/).

Next, the RA samples were stratified based on the activity of ADA in the serum samples. Samples having ADA ≤ 16 U/L were grouped as having low ADA and those having ADA ≥ 16 U/L were grouped as high ADA samples. We used a one-way analysis of variance (ANOVA) to compare differences in metabolites in healthy controls and RA patients stratified based on ADA activity. This analysis revealed 10 metabolites (Supplementary Table S2) that were significantly altered (FDR 0.25) between the three groups (Fig. 3a). Similar to the results between RA and healthy controls, both 2 aminoheptanoic acid, and hydroxyisocaproic acid were the most altered metabolites. Interestingly, inosine levels were also altered across the three groups. Pathway mapping of the differential metabolites revealed histidine metabolism, and starch and sucrose metabolism to be the most enriched pathways that distinguished the three groups (Fig. 3b). Also, RF analysis showed that both HICA and 1-methyl histidine were the most significant metabolites that accurately delineated the three groups (Fig. 3c). We observed similarly, good separation between Controls, Low ADA, and High ADA RA patients by PLS-DA method (Fig. 3d). Cross validation (Fig. 3e) resulted in good accuracy at R^2^ = 0.82917, and Q^2^ = 0.37759 for 3PCs. PLS-DA highlighted inosine, s-ribosyl-L-homocysteine, hydroxyisocaproic acid, and 1-methyl histidine as significant metabolites. The comparative analysis showed that pyruvate metabolism was common between our Low ADA metabolomic dataset and published dataset by Li et al., (Fig. 3f). The overlapping pathways of our high ADA metabolomic dataset with all the three published metabolomic datasets include alanine, aspartate and glutamate metabolism and aminoacyl-tRNA biosynthesis. The overlapping pathways of our high ADA metabolomic dataset with the studies by Li et al., and Zhou et al., are phenylalanine, tyrosine and tryptophan biosynthesis, phenylalanine metabolism, tyrosine metabolism, arginine biosynthesis, butanoate metabolism and arginine and proline metabolism. Histidine metabolism was common between our High ADA dataset and study of Li et al., and Madsen et al. The overlapping pathways of our high ADA metabolomic dataset with published datasets of Zhou et al., and Madsen et al., include purine metabolism, glycerolipid metabolism, cysteine and methionine metabolism, pentose phosphate pathway and glycine, serine and threonine metabolism. The overlapping pathways of our High ADA metabolomic dataset with Li et al., are Pyruvate metabolism and Citrate cycle (TCA cycle). The overlapping pathways of our High ADA metabolomic dataset with Zhou et al., are Ubiquinone and other terpenoid-quinone biosynthesis and Synthesis and degradation of ketone bodies (Fig. 3g). The list of significant pathways of individual studies by Li et al., Zhou et al., and Madsen et al., are given as a Supplementary Table S3.

A comparison of RA patients with low ADA versus high ADA revealed elevated levels of fructose-6-phosphate and hexose-6-phosphate (Supplementary Fig. S5) at FDR: 0.25 (Supplementary Table S4), among which fructose-6-phosphate accurately separated the two groups of patients in an RF analysis. t-test analysis of healthy controls with RA patients with Low ADA and High ADA revealed 7 and 32 metabolites respectively (Supplementary Tables S5 and S6 and Supplementary Figs. S6, S7a,b).

### Comparative analysis of deregulated pathways in our high and low ADA metabolomic datasets with high and low ADA expressing transcriptomics data of RA patients

To further validate our metabolomics data of High ADA and Low ADA RA patients, we compared their metabolomic pathways with the high ADA expressing PBMCs (GSE15573) and low ADA expressing synovial macrophages (GSE97779 and GSE10500) GEO datasets. We observed that our High ADA metabolomics dataset had 8 pathways in common with high ADA expressing PBMCs dataset (GSE15573), while only one pathway each was common with low ADA expressing synovial macrophage dataset (GSE97779) and with another low ADA expressing synovial macrophage dataset (GSE10500). Whereas our low ADA metabolomics dataset showed only one pathway common with both the high ADA expressing GEO dataset GSE15573 and low ADA expressing GEO dataset GSE97779 and no pathway for GSE10500 GEO dataset. (List of common pathways are provided in the Supplementary Fig. S8).

### Comparative analysis shows deregulated metabolomic pathways in our RA patient cohort are modulated by biologics and DMARDs from published literature

To understand the impact of the treatment with biologics and DMARDs on RA, we analyzed two published serum metabolic datasets of RA patients treated by Rituximab, and MTX. Metabolites belonging to pathways such as Glycerolipid metabolism, Phenylalanine and Tyrosine metabolism, Purine metabolism were elevated in our RA serum metabolomics dataset and were reduced in levels in RA patients who underwent rituximab treatment from the previous studies. Further, we compared with our High ADA metabolomics dataset with datasets of biologics and DMARDs treatment. Our analysis shows that the metabolites belonging to pathways such as Arginine biosynthesis, Arginine and Proline Metabolism, Betaine Metabolism, Carnitine synthesis, Galactose metabolism, Glutamate metabolism, Glycerolipid and Glycerophospholipid metabolism, Oxidation of branched chain fatty acids, Phenylalanine and Tyrosine metabolism, Valine, leucine, and Isoleucine degradation were common among the datasets and showed reduced levels in RA patients treated with rituximab and MTX. The Low ADA group did not show any significant changes when compared to RA patients under treatment. (Supplementary Fig. S9).

### Comparative analysis of metabolic pathways in RA patients shows an overlap of metabolic pathways from gene expression data sets in the GEO database

**Figure 4:**
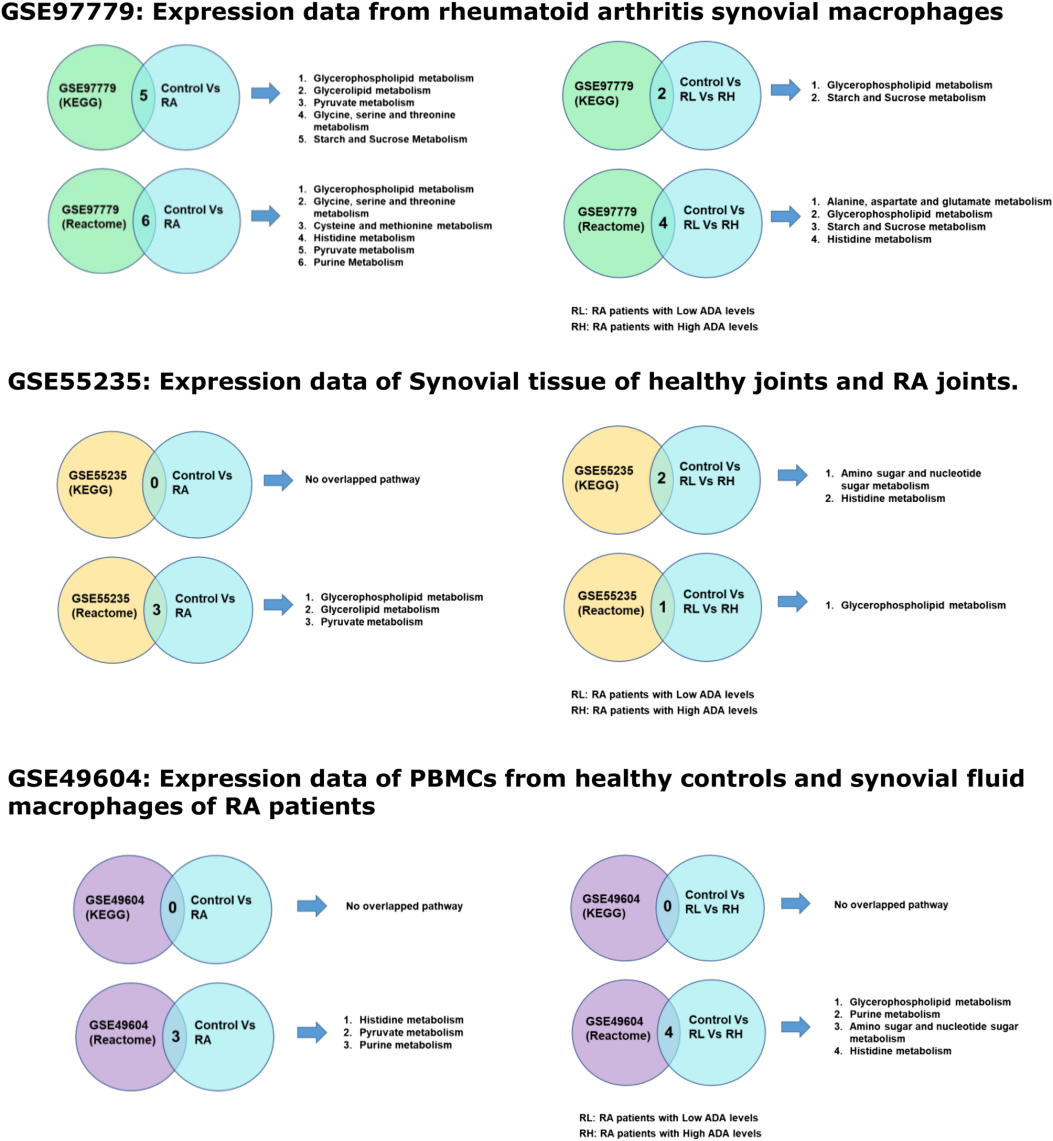
Correlation of Pathway analysis by ClueGO of GEO datasets—GSE97779, GSE55235 and GSE49604 with significant pathways from metabolomics data showed common pathways that has implications in pathophysiology of RA.

Gene expression analysis of GEO datasets related to RA and in vitro mechanistic studies were carried out using ClueGO, a Cytoscape plugin a bioinformatic tool for network analysis. We performed gene expression analysis of GSE97779 (RA synovial macrophages), GSE49604 (synovial macrophages in patients with RA and peripheral blood mononuclear cells (PBMCs) from healthy controls), GSE55235 (synovial tissue from RA joint and healthy joint). We observed a correlation between the differential metabolic pathways (Fig. 4) in serum metabolomics of RA patients and healthy controls; the transcriptomics data from gene expression datasets curated from the GEO OMNIBUS database. Predominant pathways are Glycerophospholipid metabolism and Histidine metabolism which are known to be involved in ROS generation and tissue repair.

### Effect of ADA on the differentiation of cells

**Figure 5:**
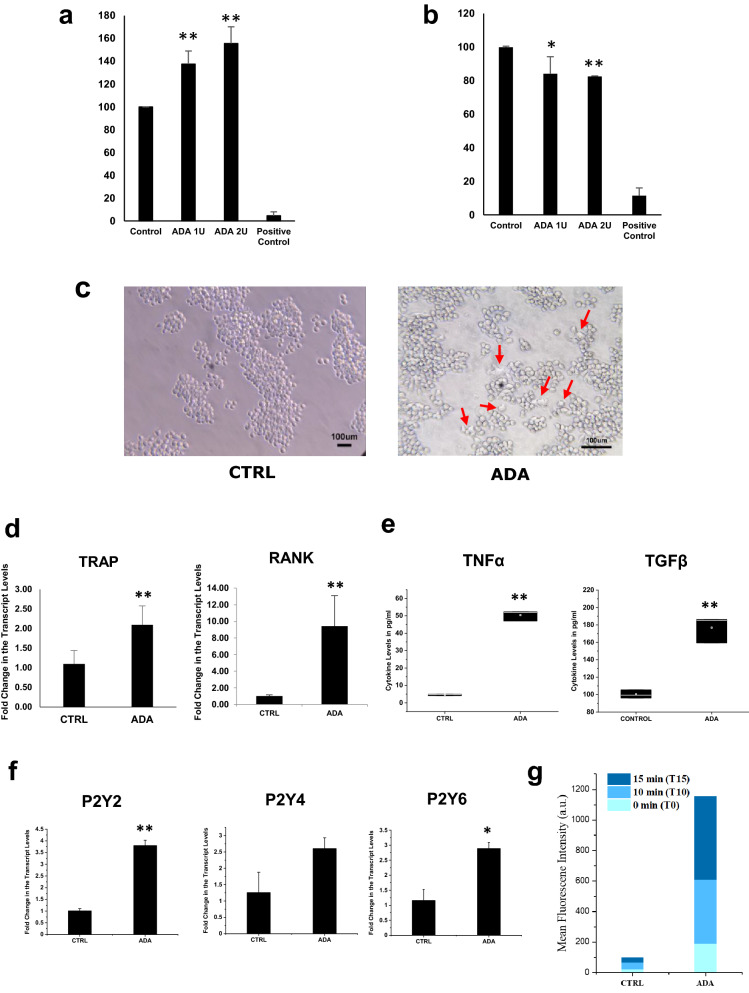
ADA is involved in joint remodeling in rheumatoid arthritis—in vitro studies to investigate the role of ADA in joint remodeling in the affected joint. (**a**) Increased proliferation of Synoviocytes, (**b**) Decreased cell viability in primary chondrocytes. (**c**) Increased Osteoclast differentiation of macrophages and (**d**) qPCR analysis showing the expression of osteoclast markers after 72 h exposure to ADA. (**e**) Increased cytokine levels in the spent medium after 72 exposure to ADA. (**f**) P2Y_2_, P2Y_4_, and P2Y_6_ receptor expression in macrophages after 72 h of ADA exposure. (showing *representing a p value of ≤ 0.05, and **representing a p value of ≤ 0.005). (**g**) Increased ROS in macrophages after ADA exposure (p values of ADA to CTRL on all time points are ≤ 0.005). All the plots were made using Origin 2020.

The effect of ADA on cell death or proliferation of primary chondrocytes and a synovial fibroblast cell line was tested using the MTT assay. Treatment with ADA led to significantly increased cell death of primary chondrocytes while it led to the proliferation of the HIG-82 synovial fibroblast cell line (Fig. 5a,b). Exposure of RAW 264.7 cells to ADA for 72 h resulted in the formation of multinucleated cells with rough margins, resembling osteoclasts (Fig. 5c), and elevated levels of osteoclast markers such as TRAP and RANK (Fig. 5d). TRAP enzyme activity was also significantly higher in cell lysates from ADA-treated RAW 264.7 cells when compared to untreated cells (controls).

As ADA is known to induce inflammation, we estimated the levels of inflammatory cytokines produced by RAW264.7 cell lines exposed to ADA. Treatment with ADA led to a significant increase in the secretion of the cytokines TNFα and TGFβ (Fig. 5e). As purinergic signaling is known to be activated by ADA, we evaluated the effect of ADA on the levels of expression of P2 receptors. P2Y_2_, P2Y_4_, and P2Y_6_ showed increased expression with ADA (Fig. 5f). The inflammatory response has also been reported to induce increased production of reactive-oxygen-species (ROS) in the cells present in the joints^31^. We observed that treatment with ADA led to a significant increase in the production of ROS in RAW264.7 cells (Fig. 5g).

**Figure 6:**
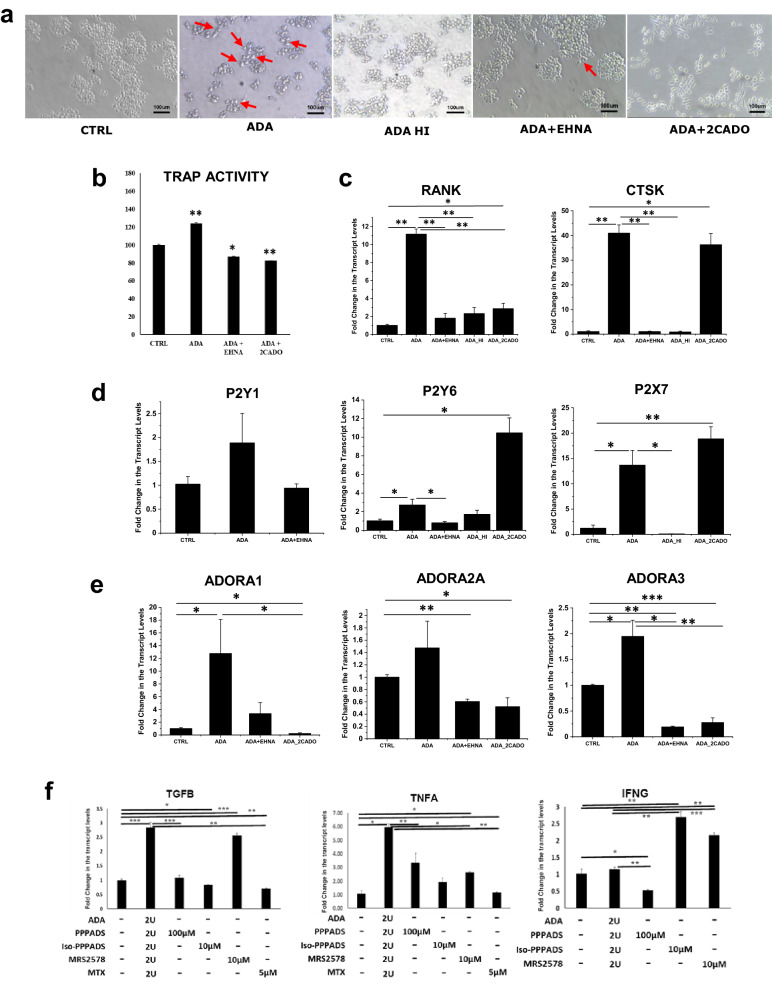
ADA enhances adenosine signaling and purinergic receptor signaling in rheumatoid arthritis. (**a**) Osteoclast differentiation is reduced after exposure to EHNA (ADA blocker) and 2CADO (adenosine analog) in macrophages. (**b**) Reduced TRAP activity after the treatment with EHNA and 2CADO confirming the role of ADA in osteoclast differentiation. (**c**) Decreased expression of osteoclast markers CTSK and RANK in macrophages after exposure to EHNA and 2CADO. (**d**) Decreased expression of P2Y receptors after exposure to EHNA and 2CADO along with ADA shows involvement of P2Y receptors in osteoclast differentiation. (**e**) Enhanced Adenosine signaling with the exposure of ADA in macrophages leading to increased expression of adenosine receptors. (**f**) Decreased expression of inflammatory genes after exposure to P2 receptor antagonists in the presence of ADA (showing *representing a p value of ≤ 0.05, and **representing a p value of ≤ 0.005, ***representing a p value of ≤ 0.0005). All the plots were made using Origin 2020.

The formation of multinucleated cells from macrophages (RAW 264.7 cells) and the increased expression of osteoclast markers brought about by ADA could be inhibited by co-treatment with erythro-9-(2-hydroxy-3-nonyl) adenine (EHNA), an ADA inhibitor; this was also accomplished by co-incubation with 2-chloro adenosine (2CADO), an analog of adenosine that does not undergo deamination by ADA. Heat-inactivated ADA (ADA HI) did not induce osteoclastogenesis in RAW 264.7 cells confirming that ADA activity plays an essential role in osteoclast differentiation (Fig. 6a). We observed a similar trend in the TRAP activity of the cell lysates of RAW 264.7 cells in the above-mentioned conditions, with an increase in TRAP activity with ADA treatment (Fig. 6b).

qPCR studies of the osteoclast markers in the ADA treated macrophages showed increased expression of RANK and CTSK and the expression was low in ADA + EHNA, ADA HI, and ADA + 2 CADO treatment (Fig. 6c). P2 receptors P2Y_6_ and P2X_7_ showed increase after ADA exposure and significant reduction was observed after the treatment with EHNA and ADA HI when compared to ADA treatment (Fig. 6d). Adenosine signaling also has an important role in osteoclast differentiation-related both in its activation and inhibition. qPCR studies on RAW 264.7 cells treated with ADA showed significant upregulation of expression of A1 and A3 receptors; no significant changes were observed in the levels of A2A receptors. Levels of A1, A2A, and A3 receptors were significantly reduced in the presence of EHNA (Fig. 6e).

Previous studies have implicated a role for ADA in inflammation^6,32^. Since P2 receptors are upregulated as a consequence of ADA treatment we probed if ADA induced inflammation is dependent on P2 receptors. RAW 264.7 cells were treated with broad spectrum P2 receptor antagonist such as pyridoxalphosphate-6-azophenyl-2′,4'-disulfonic acid (PPADS), or iso-PPADs, and MRS2578 (P2Y_6_ selective) along with ADA. RAW 246.7 cells treated with PPADS or iso-PPADS, or MRS2578 in presence of ADA showed significant reduction in expression of inflammatory cytokines such as TGFβ, and TNFα, when compared to ADA alone. But, IFNγ showed increased expression when treated with iso-PPADS and MRS2578 (P2Y_6_i) when compared to ADA alone. MTX treatment in presence of ADA also showed significant reduction in expression of cytokines such as TGFβ and TNFα compared to both ADA and untreated cells (Fig. 6f).

### Transcriptomic analysis of High ADA expressing cells shows increased expression of P2 receptors, Inflammatory cytokines and osteoclast markers

**Figure 7:**
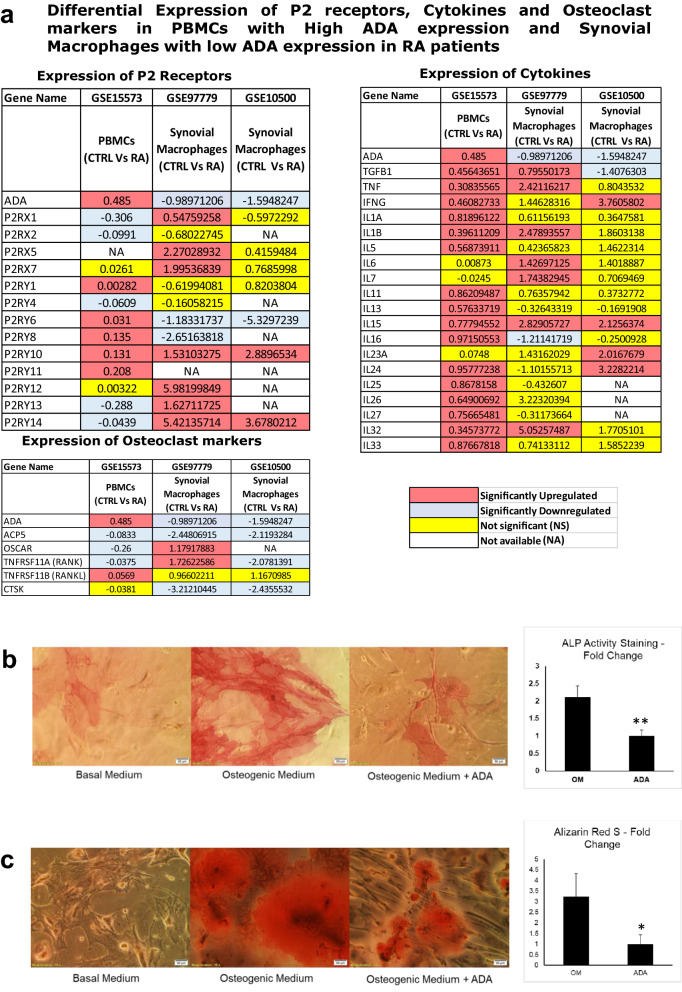
(**a**) Comparative transcriptomics data analysis of High and Low ADA expression datasets showing differential gene expression of P2 receptors, inflammatory cytokines and osteoclast markers in PBMCs and synovial macrophages respectively. (**b**) ADA treated MSCs in osteogenic medium showed significantly reduced differentiation to osteoblasts compared to those treated with osteogenic medium alone as evident from ALP staining (OM = Osteogenic Medium; ADA = Osteogenic medium with ADA). (**c**) Alizarin Red S staining shows reduced mineralization in osteoblasts differentiated from MSCs in the osteogenic medium with ADA compared to osteogenic medium alone (OM = Osteogenic Medium; ADA = Osteogenic medium with ADA). (showing *representing a p value of ≤ 0.05, and **representing a p value of ≤ 0.005). All the plots were made using Origin 2020.

We further validated the in vitro experiments on RAW 264.7 cells, showing elevated cytokine expression in the presence of ADA is of consequence in vivo. For this, we compared the transcriptomic datasets of PBMCs (GSE15573) with significantly upregulated ADA expression and synovial macrophages (GSE97779 and GSE10500) with significantly downregulated ADA expression. Our comparative analysis shows that the significantly higher ADA expressing PBMCs had increased expression of P2 receptors and cytokines compared to low ADA expressing synovial macrophages. In particular, the P2Y receptors such as P2Y_1_, P2Y_6_, P2Y_8_, P2Y_10_, P2Y_11_, inflammatory cytokines such as TGFβ1, TNF, IFNγ, IL1α, IL1β, IL5, IL11, IL13, IL15, IL16, IL24, IL25, IL26, IL27, IL32, & IL33 and some osteoclast markers were elevated in the high ADA expressed dataset (Fig. 7a).

### ADA impairs differentiation of mesenchymal stem cells to osteoblasts and mineralization

RA is also associated with reduced mineralization and erosion of bone^21^. Having examined ADA-mediated osteoclastogenesis, we investigated the effect of ADA on the differentiation of mesenchymal stem cells (MSCs) into osteoblasts and mineralization in osteoblasts. ADA inhibited the differentiation of MSCs into osteoblasts in osteogenic media as is evident from cell morphology and significantly reduced ALP staining (Fig. 7b). ADA also induced a significant reduction in mineralization as is evident from alizarin staining (Fig. 7c). These experiments show that ADA reduces the differentiation of MSCs into osteoblasts as well as mineralization in them.

## Discussion

In the current study, we observed increased serum levels of ADA, a marker of inflammation, in RA patients as has been observed in previous studies^10,33^. In particular, the activity of ADA 2 isoenzyme was found to be higher compared to ADA 1. Previous studies also reported elevated levels of ADA 2 in RA^32,34^. Increased activity of ADA in serum can shift the immunologic balance towards a pro-inflammatory bias. Inhibiting the ADA activity can help in reduction of systemic inflammation and thus the disease activity^35^. Treatment of RA patients with a combination of methotrexate and tripterygium glycosides tablets (TGTs), an inhibitor of ADA showed improvement in disease activity. The TGTs which inhibit ADA^36^ helps to maintain the increased extracellular adenosine levels due to the activity of methotrexate. Thus, the ADA can be an important role player in driving the inflammation. We also observed a significant increase in levels of the pro-inflammatory cytokines TNFα, IL-17 and IFNγ in the serum of RA patients as reported earlier^31,37,38^. Increased levels of TNFα can lead to differentiation of macrophages to osteoclasts^39^, and bone resorption^40^ in affected joints. TGFβ was also found to be elevated in our patient cohort. TGFβ also induce osteoclastogenesis and bone resorption^41^. The serum of RA patients also showed increased levels of soluble RANKL, a major role player in osteoclast differentiation.

Our High ADA patient cohort exhibited significantly higher levels of inflammation and cytokine expression. Consistent with this, our in vitro experiments with ADA treated RAW 264.7 cells show upregulated inflammatory cytokines. The upregulated cytokines could be inhibited by broad spectrum P2 receptor antagonists, P2Y_6_ antagonist and MTX. Previous studies have shown that P2 receptor antagonists attenuate inflammation in macrophages treated with LPS^42^.

To further validate, our in vivo and in vitro datasets of elevated cytokines in presence of ADA, we analyzed transcriptomic data of RA PBMCs with significantly higher ADA and RA synovial macrophages with significantly lower expression of ADA. Our results show elevated levels of many P2 receptors, inflammatory cytokines in the high ADA PBMCs dataset compared to synovial macrophages. Previous studies have shown that ADA is associated with inflammatory cytokines^35^. IL-1β and IL17A are found in the microenvironments of RA joint. Both work together to enhance the expression of pro-inflammatory cytokines such as TNFα, and IL6 and then cartilage degrading enzymes such as Matrix metalloproteinase 13 (MMP13)^43^. IL5 is known to contribute to the chronic inflammation by regulation of the B cell and eosinophil functions^44^. IL-11 is released more by the RA fibroblasts, and the macrophages in the RA synovial lining. Binding of IL-11 to IL-11Rα enhanced the fibroblast infiltration into RA affected joint, and also affects the cell proliferation in the synovium^45^. IL-11 is also reported to contribute to the osteoclast formation directly without RANKL involvement^46^. IL-15 is also a multifunctional cytokine with roles in immune cell activity, proliferation and cell viability. IL-15 and RANKL mutually induce osteoclast differentiation from macrophages and has a significant role in bone destructive diseases^47^. IL-24 along with IL-20 are involved in radiological progression of bone destruction that is manifested through RA autoantibodies^48^. IL-26 is new member of IL-20 cytokine family found to be more in the affected RA joint^49^. IL-32 is reported to enhance osteoclast differentiation and promote expression of osteoclast markers such as OSCAR, CTSK and NFATc1^50^. All these cytokines (IL-1β, TNFα, IL5, IL-11, IL-15, IL-24, IL-26, IL-32) that contribute to the RA pathophysiology were increased in the RA PBMCs dataset with significantly high ADA expression compared to synovial macrophages with insignificant ADA expression compared to control.

Chronic inflammation in the joints in RA can shift bone remodeling towards a more destructive pattern associated with increased expression of cytokines, leading to erosion of joints by stimulation of osteoclast differentiation^51^ and synovial proliferation^52^. To understand the role of ADA in joint erosions, we evaluated the SHARP scores of patients with RA, based on their ADA levels. However, we found very low correlation between ADA levels and SHARP scores. We presume that this might be due to membrane bound isoform of ADA^16,53^ which might be active in RA patients as our metabolomic data shows inosine levels were high even in the low ADA patients. Though there is a low correlation of erosion scores to high ADA levels, a study by Hameed et al.^54^, also showed ADA as a useful marker for understanding the RA disease activity by incorporating along with the DAS28 scoring. However, the data of consumption of methotrexate in RA patients with erosions will be critical to correlate ADA levels with erosions. Future studies on a larger cohort in this context will help to shed light on the role of ADA in joint erosions.

Cytokines and metabolic intermediates produced by immune cells during the inflammatory process play an important role in disease progression in RA^55^. ADA may bring about inflammation by inducing metabolic changes that support the process of osteoclast differentiation leading to bone resorption and synoviocyte proliferation. However, the reason for the increased inosine levels in both low and high ADA sets warrants further investigations. Several studies have explored the relationship between inflammation, and metabolism in the context of treatment and outcomes of RA^56,57^ as well as disease activity in RA^58^. The current study is the first to describe altered metabolism in serum samples of RA patients based on ADA activity.

Previous studies on metabolomics done in RA patients by mass spectrometry have shown that fatty acids^59^, histidine, and guanidoacetic acid^58^ and elevated levels of sphingomyelins and ceramides^60^ are associated with disease progression in RA. In the current study, we found altered levels of the metabolites involved in glucose metabolism, glycerophospholipid metabolism, phospholipid biosynthesis, histidine metabolism, pyruvate metabolism. Active processes such as osteoclast activity leading to bone and cartilage resorption, synoviocyte proliferation with infiltration, and multiplication of immune cells leading to pannus formation increase the energy demand and thus alter cellular metabolism in the affected joint. Glucose metabolism is a major contributor to the release of many intermediates that have diverse roles in different metabolic pathways^61^. The differentiation of macrophages to the M1 phenotype also upregulates aerobic glycolysis and impaired oxidative phosphorylation^62^. Our data on targeted metabolomics in RA patients with high ADA levels showed increased levels of metabolites involved in glucose metabolism. Altered glycerophospholipid metabolism can result in increased production of ROS, as reported previously^63^, and can eventually lead to increased expression of pro-inflammatory cytokines and osteoclastogenesis^64^. We found low levels of acetylcholine in the serum of RA patients; acetylcholine is involved in glycerophospholipid metabolism and may indicate bone loss as previous studies have reported that acetylcholine directs the migration of osteoblast progenitors from bone marrow thereby assisting bone formation^65^.

We also observed low levels of the histidine derivative 1-methyl-histidine. Histidine, a semi-essential amino acid, and its derivatives are needed for tissue repair and cellular growth^66^. Low levels of 1-methyl histidine may lead to increased bone resorption. Similarly, Hydroxyisocaproic acid (HICA) which is known to increase bone mass and strength^67^ was found in low levels in RA patients indicating a loss of bone and connective tissue.

Our metabolomics data from RA patients with high ADA activity showed alterations in metabolites belonging to alanine aspartate and glutamate metabolism, arginine biosynthesis, citrate cycle (tricarboxylic acid (TCA) cycle), butanoate metabolism, starch and sucrose metabolism, and pyruvate metabolic pathways. Consistent with this, high ADA sets are distinguished from Low ADA sets in a random forest analysis by hexose-phosphate and fructose-6-phosphate. Elevated levels of succinate as well as starch and sucrose metabolism may potentiate osteoclastogenesis thus enhancing disease severity^68,69^. Succinate enhances hypoxia by inhibiting the prolyl hydroxylase domain proteins that are involved in the degradation of hypoxia-inducible factor (HIF)-1 alpha, leading to HIF-1 activation^70^. Succinate is also involved in boosting the levels of inflammatory cytokines like TNFα and IL-1β^71^. The elevated cytokine profile in high ADA sets complied with the expected results of immune metabolism and inflammation.

Further to validate if our findings have broader implications, we compared our High ADA and Low ADA metabolomics datasets with the transcriptomic datasets with high (GSE15573) or low (GSE97779 and GSE10500) expression of ADA. Our results show that metabolic pathways of High ADA dataset significantly overlapped with high ADA expressing transcriptomics dataset.

Interestingly, inosine, a product of ADA activity was shown to be higher in levels in RA patients and the higher levels appear to reflect our biochemical data on increased ADA activity in the serum of RA patients. We also detected increased levels of phenylalanine as reported previously^72^; dysregulated phenylalanine metabolism is associated with immune activation or inflammation^73^. RA patients also showed increased levels of 2-aminoheptanoic acid, 2-aminooctanoic acid, 2-hydroxypyridine, shikimate, s-ribosyl-homocysteine, and phenylalanine and also decrease in deoxy carnitine, cystathionine involved in methylation, and lactate. The functional role of the metabolites 2-aminoheptanoic acid, 2-aminooctanoic acid, and 2-hydroxypyridine in the context of bone remodeling and inflammation has yet to be explored. Our metabolomic analysis shows a distinct metabolic signature in RA patients with high ADA activity and low ADA activity when compared to healthy controls; altered metabolic levels of hydroxyisocaproic acid, 1-methyl histidine, inosine, fructose-6-phosphate, and hexose phosphate can be added to the existing biochemical tests as markers that can be used as indicators of increased disease activity in RA patients.

To further corroborate the findings of our metabolomic analysis, we compared the pathways obtained in our metabolomic analysis with those obtained from analysis of metabolomics datasets from literature. We observed significant overlap between our datasets and those obtained from literature^72,74,75^ independent of RA patient cohorts and study setting.

To further understand the mechanistic aspects of biologics and DMARDs, we re-analyzed the published metabolomic datasets. Comparative analysis showed that metabolomic pathways deregulated in our RA patient cohorts were modulated by biologics and DMARDs treatments as evident from the published literature^76,77^. Previous studies have also shown modulation of inflammatory cytokines after treatment with biologics and DMARDs in RA patients^78^. Our study shows ADA induced inflammation in RAW264.7 cells could be reduced by treatment with MTX (DMARD). Taken together our results show biologics and DMARDs could mitigate joint destruction in RA.

Bone remodeling is a dynamic process in which multiple mechanisms operate. Bone formation and destruction are controlled by the synchronized activity of osteoblasts and osteoclasts. Osteoblasts form the bone by secretion of matrix proteins which make up the osteoid, where deposition of hydroxyapatite leads to bone mineralization^79^, while osteoclasts destroy the bone matrix and form pits on bone surfaces leading to its resorption. Our mechanistic studies, for the first time, show that direct exposure of macrophages to ADA leads to their differentiation into osteoclasts; this response was inhibited by EHNA, a potent inhibitor of ADA, confirming the direct effect of ADA in osteoclast differentiation. Adenosine signaling and purinergic signaling contribute to bone remodeling^80,81^. Adenosine receptors, especially the A1 receptor, play important roles in osteoclast differentiation^82,83^. Our qPCR analysis shows that exposure to ADA potentiates the expression of the A1 receptor and forms multinucleated cells to with increased TRAP activity whereas treatment with heat-inactivated ADA and the ADA along with its inhibitor EHNA shows a decreased expression of the A1 receptor suggesting that ADA can enhance the osteoclastogenesis through the A1 receptor. No change in the expression levels of A2A receptor was observed on ADA treatment and A2A expression is reported to have an opposite effect, inhibiting osteoclastogenesis^84^. The A1 and A3 receptors are found to be expressed in most of the tissues. A3 receptor expression was reported to be increased in RA PBMCs^85^ and similarly we could see a significant increase in the expression of these genes in macrophages exposed to ADA in our study. The increased expression of A1 and A2 in RAW 264.7 cells on treatment with ADA was found to be significantly reduced on treatment with ADA blocker EHNA, and also 2CADO. Overall, our experiments show that ADA can independently modulate osteoclastogenesis.

P2 receptors contribute to the formation of multinucleated osteoclasts by the fusion of macrophages^86^ and their activity through purinergic signaling. Our qPCR analysis revealed that exposure of macrophages to ADA results in increased expression of P2Y_1_, P2Y_4_, and P2Y_6_ receptors known to potentiate proliferation and differentiation of osteoclasts^87^. P2Y_1_ and P2Y_2_ are known to increase the rate of proliferation of osteoblasts, as reported earlier^88^; our demonstration of their increase in macrophages treated with ADA suggests their role in proliferation. Increased P2Y_6_ levels are known to be associated with the differentiation of osteoclasts^89^ and calcium signaling^88,90^. We observed increased expression of P2X_7_ which has been shown to increase the expression of TNFα^91^, as well as the fusion and differentiation of osteoclasts^92^. In addition to its involvement in immune responses, TNFα also initiates the differentiation of osteoclasts through the induction of RANKL and macrophage colony-stimulating factor (MCSF)^93^. In the current study, we have also shown a significant reduction in osteogenic differentiation in mesenchymal stem cells and reduced mineralization after exposure to ADA. Our study in conjunction with previous studies^7,12,33,94,95^ demonstrates a role for ADA in orchestrating joint destruction and hence could potentially be used as a marker for disease activity in RA. However, low sample size is one of the limitations of our study. Studies over a larger cohort could help develop ADA as a potential marker and therapeutic target in RA.

## Conclusions

The clinical parameters of our patient cohort were characteristic of RA. The RA patients based on ADA activity were divided into two sets (ADA high and low). ADA induced metabolic remodeling in RA and the patients with higher and lower ADA activity had distinct metabolic and cytokine profile. Metabolomic analysis shows deregulated metabolic pathways such as glycerophospholipid metabolism, histidine metabolism, purine metabolism, and pyruvate metabolism in RA. The high ADA RA patients are distinguished from low ADA RA patients by immune-metabolites like fructose 6 phosphate and hexose phosphate and elevated levels of cytokines. Further, comparative pathway analysis of our ADA High metabolomic dataset revealed a considerable overlap with high ADA expressing PBMCs dataset (GSE15573). The deregulated metabolic pathways have implications for joint biology and hence the disease. In vitro cell culture studies show ADA induce chondrocyte cell death and synoviocyte proliferation. ADA induce differentiation of RAW 264.7 cells into osteoclasts which was inhibited by EHNA or 2-chloroadenosine. ADA also induce inflammatory response in RAW264.7 cells which is inhibited by DMARDs and P2Y receptor antagonists. DMARDs treatment can modulate ADA activity as they induce anti-inflammatory effect through adenosine. DMARDS also modulate metabolic pathways deregulated in RA. PBMCs with significantly higher ADA expression show increased expression of inflammatory cytokines compared to synovial macrophages with significantly low ADA expression. ADA impaired differentiation of MSCs into osteoblasts and mineralization function. Taken together the current study demonstrates that ADA contributes to metabolic remodeling in RA and might mediate death of chondrocytes, proliferation of synoviocytes, differentiation of macrophages to osteoclasts and reduced differentiation of MSCs to osteoblasts and its function in vivo*.* Our study demonstrates ADA as a potential biomarker for monitoring disease activity and can be a potential therapeutic target in RA.

## Methodology

### Subjects

Fifty-eight patients diagnosed with RA were recruited to this study based on American college of rheumatology (ACR) Criteria (2010) from (i) the Department of Orthopedics, Sri Sathya Sai Institute of Higher Medical Sciences, Prasanthi Gram, Anantapur, Andhra Pradesh, India (ii) Sri Sathya Sai General Hospital, Puttaparthi, Anantapur, Andhra Pradesh, India (iii) Sri Sathya Sai Mobile Hospital, Anantapur, Andhra Pradesh, India and (iv) Subodaya Hospital, Tirupati, Chittoor, Andhra Pradesh, India. Twenty-two age-matched healthy individuals were recruited as controls. Patients were recruited to the study (SSSIHL/IEC/PSN/BS/2012/04) that was approved and designed as per the rules, regulations, and guidelines of the Institutional Ethics Committee (IEC) (Reg. No. ECR/616/Inst/AP/2014/RR-17), Sri Sathya Sai Institute of Higher Learning, Prasanthi Nilayam, Anantapur, India. Informed consent was obtained from all the participants including RA patients as well as healthy controls as per the rules of the IEC. Current study adhered to the tenets of the Declaration of Helsinki. Subjects ranged in age between 18 and 65 years and had no comorbidities.

A volume of 10 mL of whole blood was collected with informed consent from each patient. Serum and plasma were separated by centrifugation at 3500 rpm for 20 min. Samples were aliquoted and stored at − 85 °C until use.

### Adenosine deaminase assay

Serum samples from RA patients and healthy controls were analyzed for adenosine deaminase activity in an Olympus AU400 Biochemical Analyzer by a kit-based method (Accurex ADA 60 Infinite kit). The cut-off point for serum ADA activity was fixed at 16 U/L based on the manufacturer’s instructions. ADA activity is measured spectrophotometrically^96,97^, where ADA converts adenosine to inosine by deamination and is then converted to hypoxanthine by purine nucleoside phosphorylase (PNP). Hypoxanthine is converted to uric acid and hydrogen peroxide (H_2_O_2_) by xanthine oxidase (XOD) which eventually generates a quinone dye. The intensity of the color developed is directly proportional to the activity of the ADA in the sample.

### Statistical corrections

SPSS software (IBM SPSS Statistics Subscription Base Edition) was used for the statistical corrections or adjustment of covariates of age and gender for the clinical parameters. Value beyond the normal range is taken as abnormal (1) and within the normal range is taken normal (0) for analysis purposes. We have used general linear model and univariate analysis set to full factorial model with significance of 0.05, and confidence interval of 95% and parameter estimates with robust standard errors to ensure that impact of covariates of age and gender is reduced on the ADA activity and other clinical parameters.

### Activity of ADA isoforms (ADA 1 and ADA 2)

To estimate the ADA activity of isoenzymes, we have taken the aliquots of the same samples for which the metabolomic analysis, and cytokine assays were done previously. For estimation of ADA1 (intracellular) and ADA2 (extracellular) in serum samples of RA (n = 10) and the healthy controls (n = 9), first the total ADA activity was estimated by biochemical analyzer and then after which to the each of the same sample, a potent ADA 1 activity inhibitor EHNA at a final concentration of 25 µM was added and the ADA 2 activity was estimated after incubation^98^. The ADA 1 activity was calculated by subtracting the ADA 2 activity from the total ADA activity.

### Enzyme-linked immunosorbent assay (ELISA)

Sera were screened for anti-cyclic citrullinated protein antibodies (ACPAs) antibodies by Immucheck CCP ELISA Kit (Cat. No. IS.96.015, Immunoshop, Mumbai, India), and optical density was measured at 450 nm using the manufacturer’s protocol. Patients with a reading of > 25 RU/ml (relative units/ml) were considered positive for RA. TNFα, IFNγ, IL-6, IL-10, transforming growth factor-beta (TGFβ), and soluble RANKL (sRANKL), were analyzed spectrophotometrically in a multiplate reader (Varioskan LUX, Thermo Fisher Scientific Inc., Vantaa, Finland). TNFα (Cat. No. CHC1753), TGFβ (Cat. No. CHC1683), IFNγ (Cat. No. CHC1233), and IL-10 (Cat. No. CHC1323) (Invitrogen Cytoset Antibody Pair Kit**,** California, USA) and IL-6 (Cat. No. 900 M16) and sRANKL (Cat. No. 900 M142) (Peprotech Mini ABTS ELISA Kit, New Jersey, USA) were estimated using manufacturers’ protocols and calculated by the 4-parameter curve-fit method. Sample concentrations of cytokines were obtained by comparison with a 7-point standard curve.

### Metabolomics

#### Sample preparation

A volume of 50 µL serum from RA patients and healthy controls was combined to make a master pool of serum to run as a reference for every batch. Pooled serum was stored as 50 µL aliquots at − 85 °C for further use. Samples were prepared for metabolomics as follows: To a volume of 50 µL serum sample, 150 µL of labeled internal standards solution made with 50% methanol was added to a microcentrifuge tube and then incubated in ice for 30 min. After incubation, the samples were sonicated in a water bath sonicator (Bransonic Ultrasonic MH Cleaning Bath, Branson, Danbury, USA) for 15 min and then centrifuged at 10,000 rpm for two minutes at 4 °C. Supernatants were transferred to conditioned (filter units were spun with 50:50 methanol: water for 120 min at 4 °C at 13,000 rpm) Amicon filters (Amicon Ultra 0.5 mL filter unit, Cat. No. UFC500396, Merck Millipore, Massachusetts, USA) and centrifuged at 13,000 rpm for 2 h. A volume of 180 µL filtrate was collected from each Amicon filter unit and transferred to a fresh microcentrifuge tube for aliquoting and storing until further use. A volume of 25 µL was then used for analysis using the Agilent 6490 iFunnel triple quadrupole LC/MS system. Samples were analyzed in the mass spectrometer operated in positive and negative electron spray ionization (ESI + /−) mode. For positive ionization mode, Waters X-Bridge amide 3.5 µm, 4.6 × 100 mm column (part no. 186004868, Waters, Milford, USA) was used for separation with a mobile phase of Solvent A: grade water (Water, Optima™ LC/MS Grade, Cat. No. W6500, Fisher Chemical™, Fair Lawn, NJ, USA) + 0.1% formic acid (FA) (Formic Acid, 99.0 + %, Optima LC/MS grade, Cat. No. A117-50, Fisher Chemical, Fisher Scientific, Fair Lawn, NJ, USA) and Solvent B: 100% Acetonitrile (ACN) (Acetonitrile, Optima LC/MS grade, Cat. No. A955, Fisher Chemical, Fisher Scientific, Fair Lawn, NJ, USA) + 0.1% formic acid (FA) at a flow rate of 0.3 mL/min in a gradient of 15%:85% from 0th to 3rd min, 70%:30% from 3^rd^ to 12th min, 98%:2% from 12th to 15th min, 98%:2% from 15th to 16th min, 15%:85% from 16th to 23rd min and 15%:85% from 23rd to 28th min of solvent A and solvent B respectively. For negative ionization mode, the above-mentioned column was used and with a mobile phase of Solvent A: 20 mM Ammonium acetate (Ammonium Acetate (Optima LC/MS), Cat. No. A11450 Fisher Chemical, Fisher Scientific, Fair Lawn, NJ, USA) in water (95%) and Acetonitrile, pH 9.0 and Solvent B: 100% Acetonitrile (ACN) at a flow rate of 0.3 mL/min in a gradient of 15%:85% from 0th to 3rd min, 70%:30% from 3rd to 12th min, 98%:2% from 12th to 15th min, 98%:2% from 15th to 16th min, 15%:85% from 16th to 23rd min and 15%:85% from 23rd to 28th min of solvent A and solvent B respectively. The instrument was set to delta EMV of 400 V a capillary voltage of 3000 V, the capillary temperature of 250 °C, sheath gas heater temperature of 350 °C, sheath gas flow of 12 units, and nebulizer pressure of 20 psi. To validate the consistency and reproducibility of the analysis, three tubes of pooled serum (quality control) were also extracted and analyzed in the same way as samples by injecting them in the beginning, the middle, and at the end of the run. Three blanks (50:50—Methanol:Water) runs were injected between each sample to ensure no carryover from one sample run to the other.

#### Quality controls

The acquired metabolomics data were normalized by internal standards; L-Zeatine for the positive mode and L-Tryptophan for the negative mode of acquisition. The robustness of the data acquired over different batches of samples was verified by injecting sample replicates from previous batches. Pooled sera were used as batch controls at the beginning, middle and at the end of the batch run to avoid batch variations. Three blanks were injected to ensure there is no carryover from one sample to the next sample run.

### Statistical analysis

Abundances for each metabolite were obtained using the Agilent Mass Hunter—Quantitative analysis software (Version No. B.07.00). Integration of peaks was done based on the retention time of each metabolite. The Peak area of each metabolite was normalized using an internal standard spiked along with the samples and then log-transformed for further statistical analysis in Metaboanalyst 4.0^99^ (https://www.metaboanalyst.ca/home.xhtml) (an online tool to analyze the data from metabolomics). For identifying differential metabolites between RA patients and healthy controls, a two-way t-test analysis was done, and for analysis between healthy controls, Low ADA, and High ADA RA patients a one-way ANOVA was done with post-hoc analysis. Significant metabolites were identified based on p-value adjustment done by multiple testing at a false discovery rate (FDR) threshold of 0.25. PCA analysis was done with 5 principal components on metabolomics data with 95% confidence levels to observe the clustering or separation among samples. Later to validate the separation, PLS-DA model was administered to confirm that the separation among the sample groups is significant by tenfold cross validation (CV) and with. Receiver operating characteristic (ROC) analysis was done to evaluate the performance of the metabolite as a biomarker. The true positive rate (sensitivity) and true negative rate (specificity) were estimated at 0.25 threshold and all the metabolites were ranked based on the area under the ROC curve (AUROC) at 95% confidence with 500 bootstrappings. Random Forest (RF) analysis was done to evaluate the importance of metabolites in separating or stratifying RA patients from healthy controls based on the mean decrease in classification accuracy after 5000 permutations.

### Comparative analysis with published metabolomic datasets

Three independent serum metabolomics datasets of RA patients^72,74,75^ were taken from the published literature and the deregulated metabolite levels were compared with our metabolomics dataset of RA patients. Metaboanalyst 5.0^100^ was used for the pathway analysis of the deregulated list of metabolites. The pathways listed in all three studies were overlapped with significant pathways of our metabolomics dataset for correlation using Venn Diagram tool (http://bioinformatics.psb.ugent.be/webtools/Venn/). The tool listed out the pathways that are common to our metabolomics dataset and other published studies.

### Gene expression data set analysis

Gene expression datasets was searched from the GEO OMNIBUS public gene expression studies database (https://www.ncbi.nlm.nih.gov/geo/). GEO2R tool from GEO database was used to analyze the samples for differential gene expression. We used geo datasets such as GSE97779, GSE49604, and GSE55235 of tissues and cells involved or associated with RA and its pathology for the analysis. Later we analyzed the gene expression data with adjusted p-value less than 0.05. The gene expression data was analyzed for pathway analysis using a Cytoscape bioinformatics software^101^ with a ClueGO plugin^102^. ClueGO plugin analyzed the GEO2R output of differential gene expression data to give a network of pathways involved based on the KEGG^103^ and Reactome^104^ databases. We compared the pathways from metabolic analysis of serum RA patients and GEO datasets to arrive at overlapping pathways. We also used geo datasets of RA PBMCs with significantly increased ADA expression (GSE15573) and RA synovial macrophages with significantly low ADA expression (GSE97779 and GSE10500) to compare the differential expression of inflammatory cytokines, P2 receptors, and some osteoclast markers. The logFC values by GEO2R is expressed in Log2-fold change between healthy control and RA cohorts.

Further, we also compared the deregulated pathways of high ADA expressing (GSE15573) and low ADA expressing (GSE97779 and GSE10500) GEO dataset with our metabolomics datasets of RA patients with high and low ADA levels to identify the common pathways.

### Cell culture studies

#### Cell culture

Human primary chondrocytes (extracted the tissue from cartilage biopsy of a patient with knee replacement surgery or arthroscopic surgery), rabbit synoviocytes [HIG-82: cell line from National Center for Cell Science (NCCS)], macrophage (RAW 264.7: cell line from NCCS) cells, osteoblasts (MG63: cell line from NCCS), and mesenchymal stem cells (MSCs: bone marrow stem cells (BM-MSCs) extracted from the 6–8-week-old CD-1 female mice weighing 25–28 g) were used for the study. All the animal experiments were carried out with an approval by the Institutional Animal Ethics Committee, Indian Institute of Science, Bangalore, India. All animal protocols were in line with the guidelines for care and use of laboratory animals set by Indian National Science Academy and as per the ARRIVE guidelines. All cell lines were cultured at 5% CO_2_ and 37 °C for growth and maintenance. Primary chondrocytes were cultured in HAMS F12 medium (Cat. No. AT025, Himedia Laboratories, India) with l-glutamine, sodium bicarbonate, supplemented with 10% fetal bovine serum (FBS) of South American origin (Cat. No. 10270-106, Invitrogen, Thermo Fisher Scientific Inc. New York, USA) ascorbic acid (0.1 mg/mL), 1% antibiotic–antimycotic (ABAM) (penicillin, streptomycin, amphotericin) solution (Cat. No. 15240062, Invitrogen, Thermo Fisher Scientific Inc. New York, USA). Synoviocytes (HIG-82) were cultured in HAMS F12K (Kaighn’s Modification) (Cat. No. AT106, Himedia Laboratories, India) with L-glutamine, sodium bicarbonate, supplemented with 10% FBS, 1% ABAM solution. Macrophages (RAW 264.7 cells) and osteoblasts (MG63) were cultured in Dulbecco’s modified eagle medium (DMEM) (Cat. No. AT007, Himedia Laboratories, India) with high glucose, L-glutamine, sodium bicarbonate, and FBS. MSCs were revived in a basal medium (BM) (DMEM with 10% FBS, 1% ABAM), and after reaching 70% confluence, basal medium is replaced with osteogenic medium (OM) (basal medium with 100 µM of ascorbic acid (Cat. No. A4544, Sigma Aldrich, St. Louis, USA), 5 mM of β-glycerophosphate (Cat. No. 50020-100G, Sigma Aldrich, St. Louis, USA). The culture was allowed to differentiate over 7 days for differentiation of MSCs.

#### Cell proliferation assay

The impact of ADA on the growth of primary chondrocytes and synoviocytes, (3-(4,5-dimehylthiazolyl-2)2,5-diphenyltetrazolium bromide) was investigated in the MTT assay. Cell viability was measured by the capacity of cells to convert MTT to formazan, a purple-colored crystal, which is directly proportional to the percentage of living cells^105^. Primary chondrocytes and synoviocytes were seeded at 5 × 10^3^ cells/well and 3 × 10^3^ cells/ well respectively in a 96-well plate for the assay. After 24 h of growth, ADA was added at 1U and 2U concentrations and incubated for 72 h. Pyrrolidine di-thiocarbamate (Cat. No. RM1437, HiMedia laboratories Pvt. Ltd., Mumbai, India), a nuclear factor κB (NFκB) inhibitor, was used as positive control at 350 µM. At the end of 72 h incubation, MTT (cell culture tested, Cat. No. TC191-1G, Himedia Laboratories, India) was added in the dark at 0.5 mg/mL concentration to each well and incubated at 5% CO_2_ and 37 °C for three hours after which the medium with MTT was aspirated and 150 µL of dimethyl sulfoxide (DMSO) was added to each well. DMSO dissolves the formazan crystals and gives a purple color solution proportional to the density of living cells. The percentage of living cells was determined by measuring the absorbance of the wells at 570 nm in a multiplate spectrophotometer (Spectramax, Molecular Devices, California, USA).

#### Osteoclast differentiation

Macrophages can differentiate into osteoclasts upon exposure to factors such as RANKL^106^ and macrophage colony-stimulating factor (MCSF)^107^. To study the role of ADA in the differentiation of macrophages to osteoclasts, macrophages were seeded at 10 × 10^3^ cells/well in a 24-well plate for 24 h at which point ADA (Cat. No. LS009043, Worthington Biochemical Corporation, New Jersey, USA) and metabolites were added. After 72 h of incubation, multinucleated cells (i.e., with more than 3 nuclei), with rough margins were observed microscopically.

### TRAP activity

Functionally, the differentiation of macrophages to osteoclasts can be measured by the activity of tartrate-resistant acid phosphatase (TRAP) which is expressed by osteoclasts. Macrophages were seeded at 10 × 10^3^ cells/well in a 24 well plate and after 24 h of cell growth, cells were exposed to ADA and metabolites. After 72 h of incubation at 5% CO_2_ and 37 °C, TRAP was estimated base on the colorimetric method with p-nitrophenol phosphate (pNPP)^108,109^ in a multiplate reader (Varioskan LUX, Thermo Fisher Scientific Inc., Vantaa, Finland).

#### P2 receptor inhibitor and DMARD studies

To understand the role of P2 receptors and also MTX, a DMARD in inflammation driven by ADA, macrophages were exposed to P2 receptor antagonists and also MTX along with ADA. At seeding density of 25 × 10^3^ cells/well in a 24 well plate, and after 24 h cell growth, they were exposed to non-selective P2 receptor antagonists such as PPADS (Cat. No. ab120009, Abcam Plc. Cambridge, UK), iso-PPADS (Cat. No. sc-204019, Santa Cruz Biotechnology, Inc. Dallas, Texas, USA), a selective P2Y_6_ antagonist—MRS2578 (Cat. No. S2855, Selleck Chemicals, Houston, USA), and Methotrexate (Ipca Laboratories Ltd. Mumbai, India).

#### Quantitative PCR analysis

Osteoclasts were characterized by the expression of RANK, cathepsin K (CTSK), and TRAP. And also, to understand the role of purinergic signaling and adenosine signaling on osteoclast differentiation quantitative PCR analysis was done to estimate the change in the expression of these markers in macrophages. To understand the role of P2 receptors in the presence of ADA on inflammation, the gene expression of inflammatory cytokines such as TNFα, TGFβ, and IFNγ were estimated by qPCR. Primer sequences for AdoRA1 (forward: ATCCTCACCCAGAGCTCCAT; reverse: CGCTGAGTCACCACTGTCTT), AdoRA2A (forward: AACCTGCAGAACGTCACCAA; TGCTGATGGTGATGGCGAAT), AdoRA3 (forward: GACTGGCTGAACATCACCTACA; reverse: GTTCAGCTTGACCACCCAGAT), TNFα (forward: TCTTCTGTCTACTGAACTTCGG; reverse: AAGATGATCTGAGTGTGAGGG), TGFβ (forward: GAACCAAGGAGACGGAATACAG; reverse: GGAGTTTGTTATCTTTGCTGTCAC), and IFNγ (forward: GAAAGACAATCAGGCCATCAGC; reverse: GCATCCTTTTTCGCCTTGCT) were procured from Integrated DNA Technologies (IDT), Iowa, USA whereas TRAP (forward: GAAGAGCCTTCAAGTAAGTG; reverse: ATGAATCCATCTTCTAGGGAG), CTSK (forward: CAGAGGGTACAGAGAGATTC; reverse: TCATCATAGTACACACCTCTG), RANK (forward: GAAATAAGGAGTCCTCAGGG; reverse: TAGAATCTCTGACTTCTGCC), P2Y_1_ (forward: TTGTAACATGGTCACAAGAC; reverse: AGGTGTCTTTCAACAAAACG), P2Y_2_ (forward: GCATATGTGAGTGAAGAACTG; reverse: CATTGATGGTGCCTATTCCAG), P2Y_4_ (forward: GAACTGGAACTAAGATGGTG; reverse: GTGATGTGAATAGCAAGGAG), P2Y_6_ (forward: GGAGTTTTCAGATAAACGGTG; reverse: CTCTGGTGGCTTTTAGTTTAC), and P2X_7_ (forward: ATCGAGATCTACTGGGATTG; reverse: GTACTTGGCATATCTGAAGTTG) receptors were procured from Sigma Aldrich Chemical Pvt. Ltd., Bangalore, India. A 10 µL reaction with SYBR green dye-based PCR with ROX reference was used to quantify the expression of the genes mentioned above in a 384 well format PCR machine—Quant Studio 5, Applied Biosystems, Massachusetts, USA.

#### ROS assay

Reactive oxygen species (ROS) have been reported to enhance osteoclastogenesis in vivo^110^. To determine the role of ADA in the generation of ROS from macrophages, macrophages (RAW 264.7 cells) were seeded at 3 × 10^3^ cells/ well and ROS assay was done as described previously by Wu and Yotunda^111^. Generation of ROS was measured at time points of 0, 10 and 15 min by fluorescence emitted by a fluorescent dye 2′,7′-dichlorofluorescein diacetate (DCFDA, Cat. No. 35845, Sigma Aldrich) with excitation wavelength as 485 nm and emission wavelength as 530 nm in a multiplate reader (Varioskan LUX, Thermo Fisher Scientific Inc.).

#### Alkaline phosphatase (ALP) staining

The multipotent ability of MSCs to differentiate into osteoblasts in an osteogenic medium was assessed by the ALP staining^112–114^. After the 70% confluency was reached, cells were exposed to ADA and cultured for 7 days with regular change of media every 48 h. At the end of the 7th day, the spent medium was discarded and the cells washed twice with phosphate buffer saline (PBS). 2 mL of 4% paraformaldehyde was added, and the dish was incubated at room temperature for 20 min. After 20 min of fixation, paraformaldehyde was discarded and the cells washed thrice with 2 mL of Tris maleate buffer. 2 mL of staining solution (tris maleate buffer with 10% MgCl_2_, 0.1% naphthol AS-MS phosphate (Cat. No. N5625, Sigma Aldrich, St. Louis, USA), 0.1% fast red TR salt (Cat. No. F6760, Sigma Aldrich, St. Louis, USA) was added, and the dish was kept in dark by covering with aluminium foil. After the pink color was developed over a few minutes, the staining solution was discarded and the cells washed with 2 mL of PBS. The stained cells were imaged in bright field and phase contrast microscopes.

#### Alizarin red S (ARS) staining

ARS staining was done to estimate the mineralization of osteoblasts and to assess the impact of ADA treatment on mineralization^112,114^. After 70% confluency was reached in the osteogenic medium in a 35 mm dish, cells were treated with ADA and then cultured for 21 days with regular change of media every 48 h. The spent medium was aspirated at the end of 21 days without disturbing the monolayer of cells and washed with PBS. After removing PBS, neutral buffered formalin (10%) was added to cover the monolayer of cells. After 30 min of incubation, the formalin was removed and cells were washed with double distilled water. Distilled water was removed and ARS (Cat. No. A5533, Sigma Aldrich, St. Louis, USA) staining solution was added enough to cover the monolayer. The plates were incubated in the dark for 45 min. After the incubation, the ARS staining solution was carefully aspirated and the cells washed 4 times with distilled water. PBS was added to the plate after discarding the distilled water and the cells were observed under a microscope for calcium deposits (mineralization) with bright-orange color.

## Supplementary Information

Supplementary Information.

## Data Availability

Data is available from the corresponding author on reasonable request.
