# Supplementary material for: Adenosine deaminase modulates metabolic remodeling and orchestrates joint destruction in rheumatoid arthritis
Source: Sci Rep. 2021 Jul 23;11:15129. doi: 10.1038/s41598-021-94607-5 (PMC8302689; doi:10.1038/s41598-021-94607-5)
Supplement: Supplementary file 1 — Supplementary Information. [file 41598_2021_94607_MOESM1_ESM.pdf]

Adenosine deaminase modulates metabolic remodeling and orchestrates joint destruction in rheumatoid arthritis

Sai Krishna Srimadh Bhagavatham[1]; Prakash Khanchandani[2*]; Vishnu Kannan[1,3]; Damodaram Potikuri[4]; Divya Sridharan[5], Sujith Kumar Pulukool[1]; Ashwin Ashok Naik[1]; Rajesh Babu Dandamudi[6,7]; Sai Mangala Divi[8]; Ashish Pargaonkar[9]; Rahul Ray[2]; Saibharath Simha Reddy Santha[1]; Polani B Seshagiri[5], Narasimhan K[4]; Narsimulu Gumdal[4], Venketesh Sivaramakrishnan[1*]

[1]Department of Biosciences, Sri Sathya Sai Institute of Higher Learning, Puttaparthi, India – 515134.

[2]Department of Orthopedics, Sri Sathya Sai Institute of Higher Medical Sciences, PG, Puttaparthi, India – 515134.

[3]Current address: Department of Botany/Biotechnology, CMS College, Kottayam, India – 686001.

[4]Sri Sathya Sai General Hospital, Puttaparthi, India – 515134.

[5]Molecular Reproduction and Developmental Genetics, Indian Institute of Science, Bangalore – 560012.

[6]Previous address: Department of Chemistry, Sri Sathya Sai Institute of Higher Learning, Prasanthi Nilayam, India.

[7]Current address: Phenomenex India, Hyderabad, Telangana, India – 500084.

[8]Department of Biochemistry, Sri Sathya Sai Institute of Higher Medical Sciences, PG, Puttaparthi, India – 515134.

[9]Agilent Technologies India Pvt Ltd, Bengaluru, India - 560048.

*To whom correspondence has to be sent:

Venketesh Sivaramakrishnan s.venketesh@gmail.com, and

Venketesh Sivaramakrishnan ORCID- 0000-0003-3094-5905

Prakash Khanchandani prakash.k@sssihms.org.in

Figure S1:

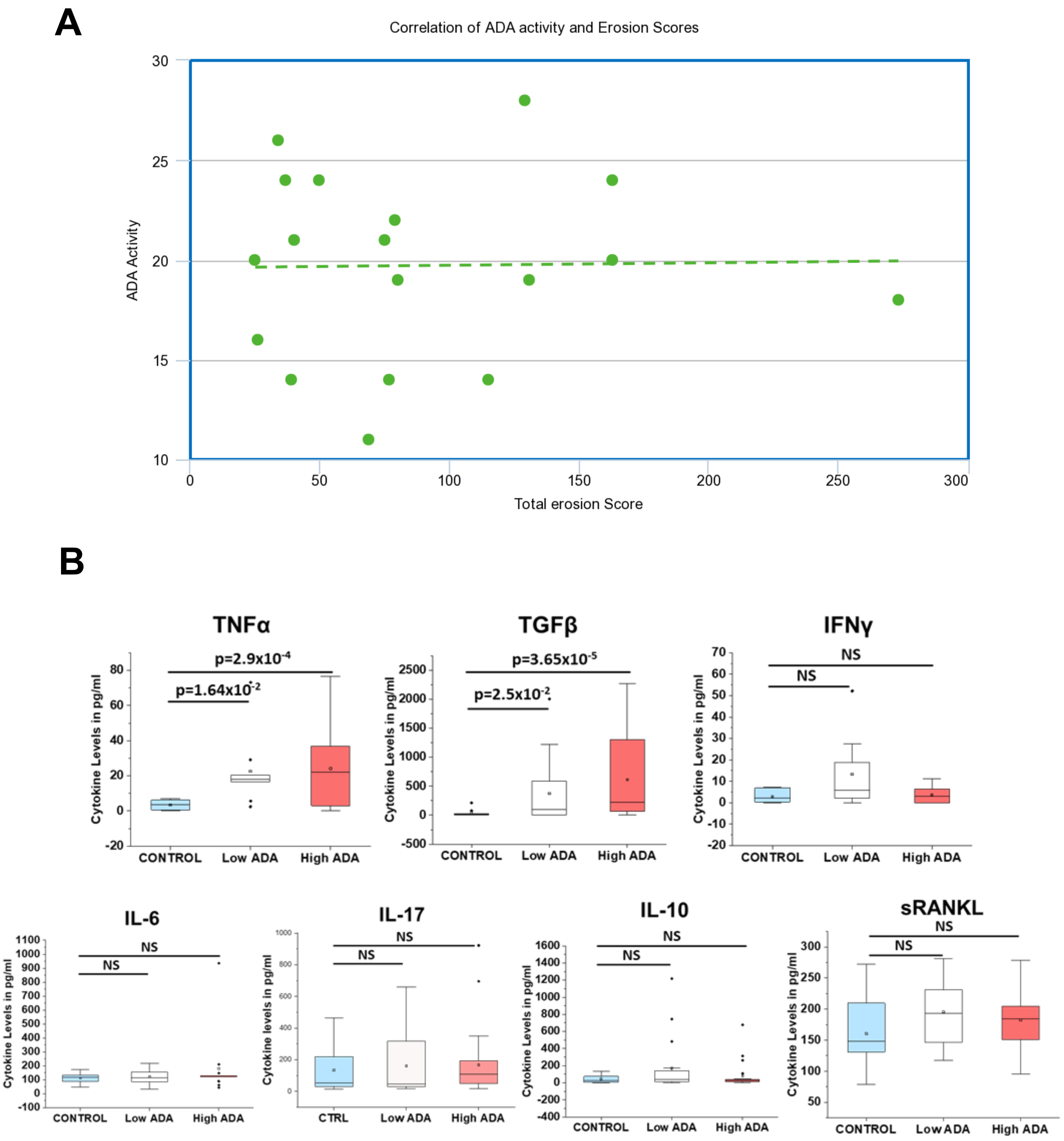

A. Correlation analysis of ADA levels with SHARP Score of RA patients **B.** T-test analysis of Cytokines of Low ADA and High ADA RA patients with healthy controls.

Figure S2: Relative abundance levels of samples after log transformation and normalization based on the internal standards in both positive and negative ionization methods.

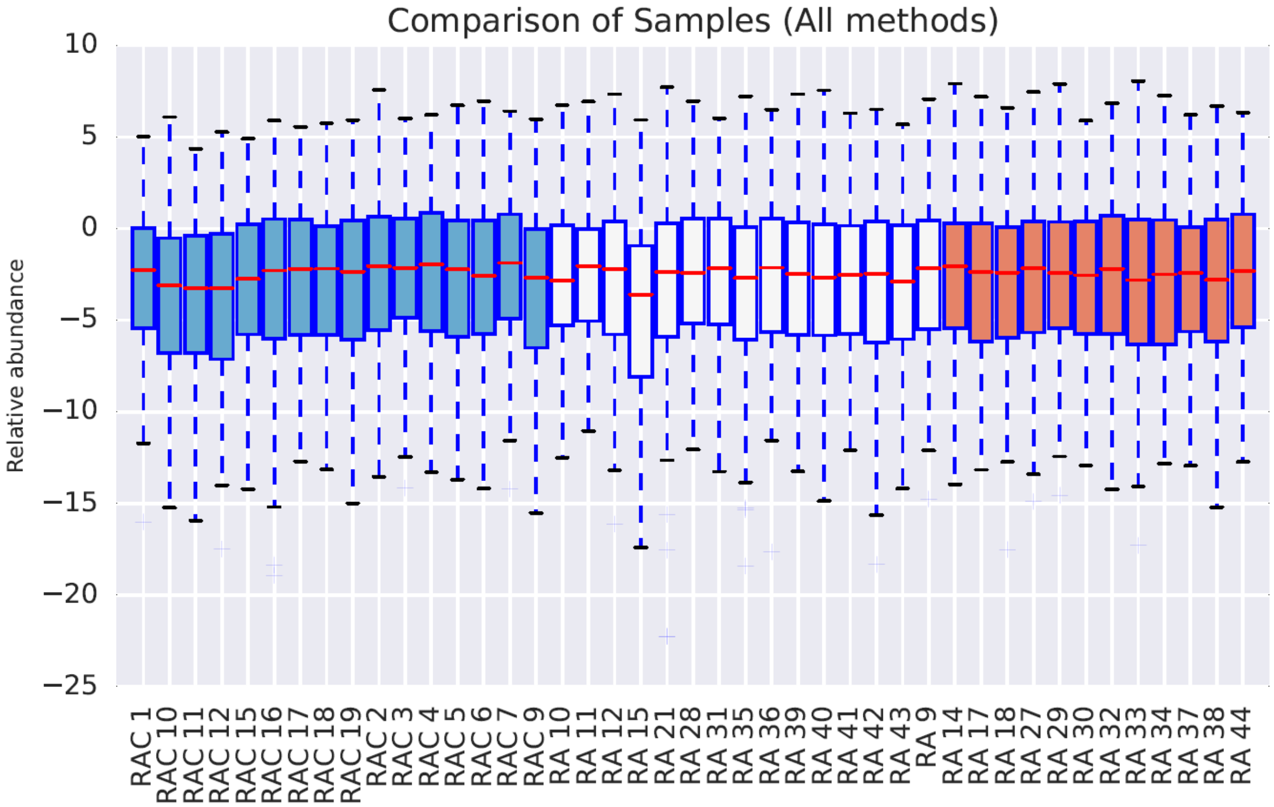

Figure S3: Correlation analysis showing good concordance among the replicates done on different days as quality control in Positive Ionization Mode.

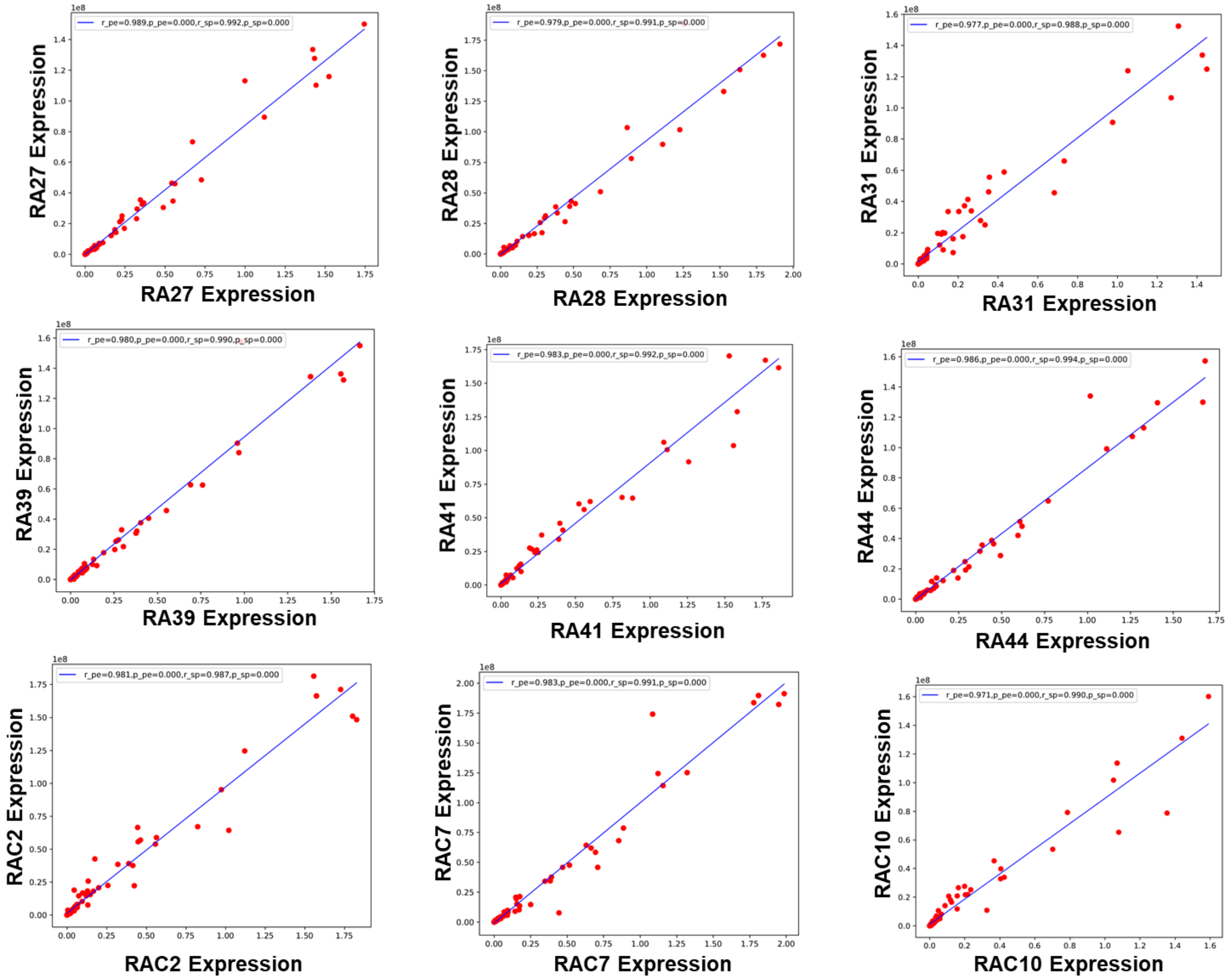

Figure S4: Correlation analysis showing good concordance among the replicates done on different days as quality control in Negative Ionization Mode.

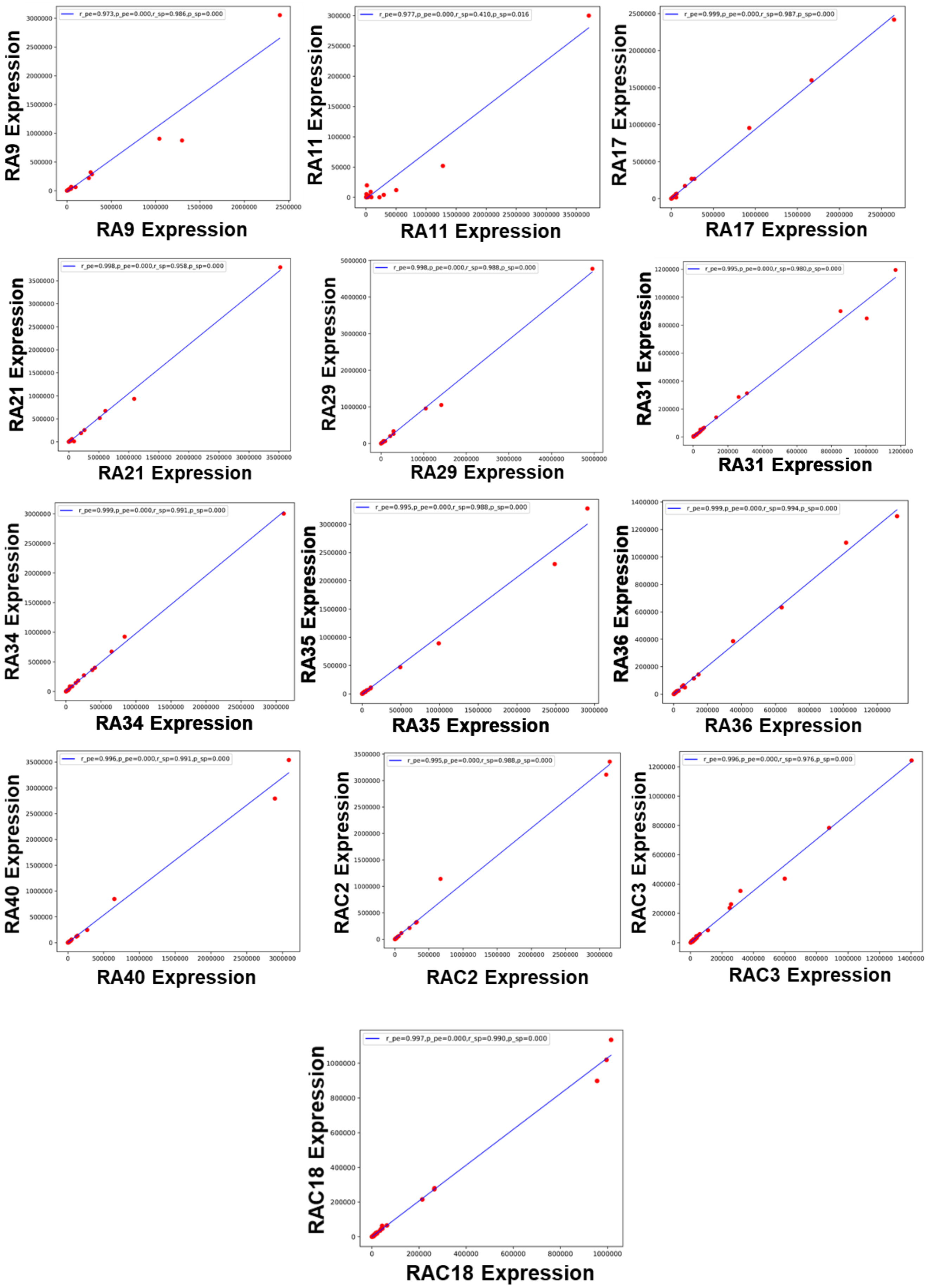

Figure S5: Targeted Metabolomics data showing altered metabolic signatures in RA patients with High ADA levels compared to Low ADA levels

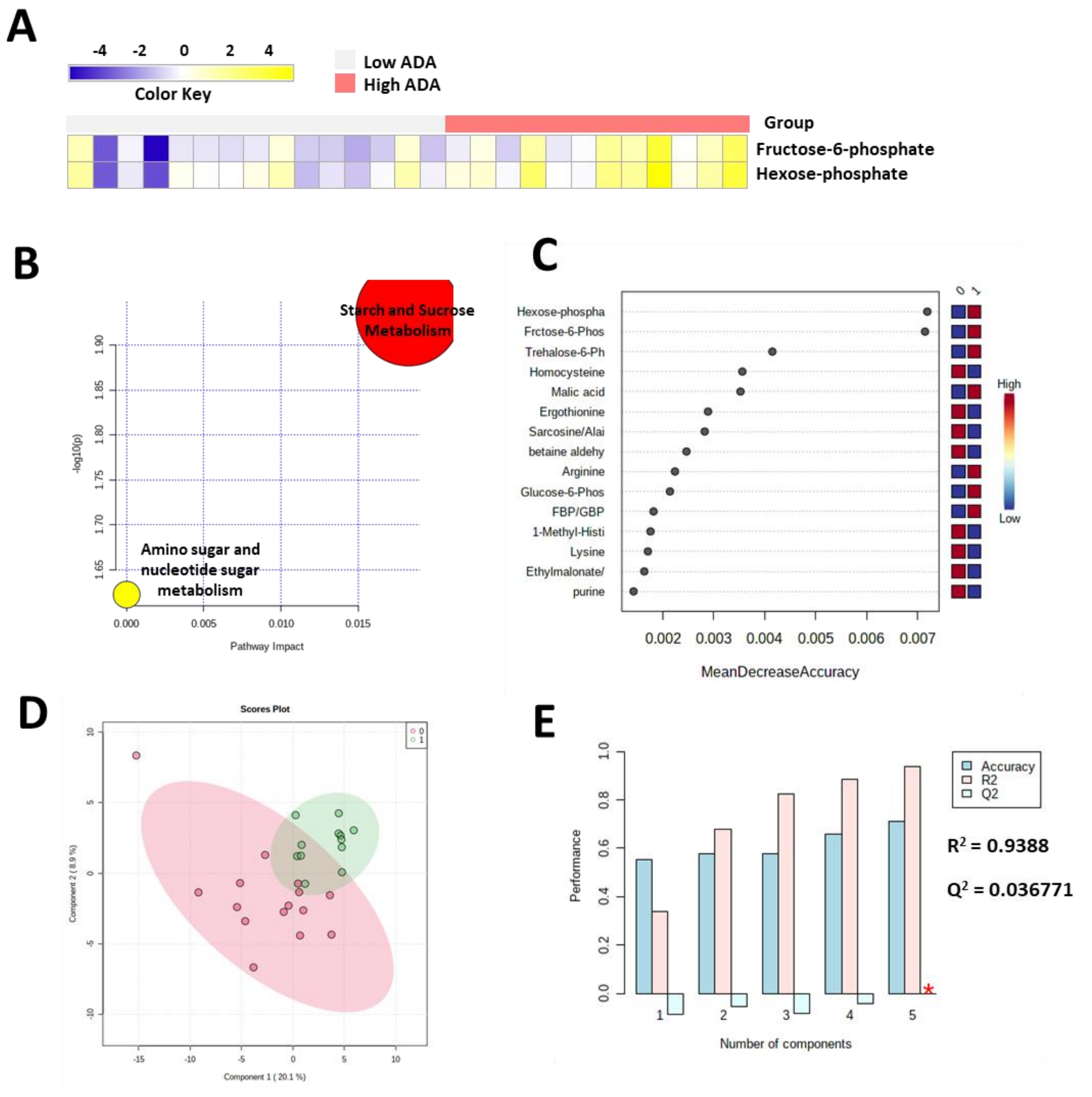

A. Heat map showing the relative levels of 2 significantly altered metabolites at FDR 0.25 with shades of yellow and blue representing the elevated and reduced metabolite levels, respectively (heatmaps were made in Microsoft Excel). **B.** Pathway analysis showing altered metabolic pathways in RA Patients with High ADA levels compared to Low ADA levels. The size of the circle represents the impact of each pathway and color represents the level of significance (highest in red to lowest in yellow). **C.** Ranking of significant metabolites based on random forest analysis for their ability to stratify RA patients with High ADA levels from Low ADA levels (ranked by the mean decrease in classication accuracy when they are permuted). **D.** PLS-DA clustering given by score plot (0=Low ADA RA patients, 1=High ADA RA patients) and **E.** cross validation of PLS-DA of RA patients with Low ADA and High ADA levels (red star indicates the best classifier based on accuracy, R^2, and Q^2 values) (Figures B, C, D, and E were generated using the web-based tool Metaboanalyst https://www.metaboanalyst.ca/).

Figure S6: Targeted Metabolomics data showing altered metabolic signatures in RA patients with low ADA levels compared to healthy controls

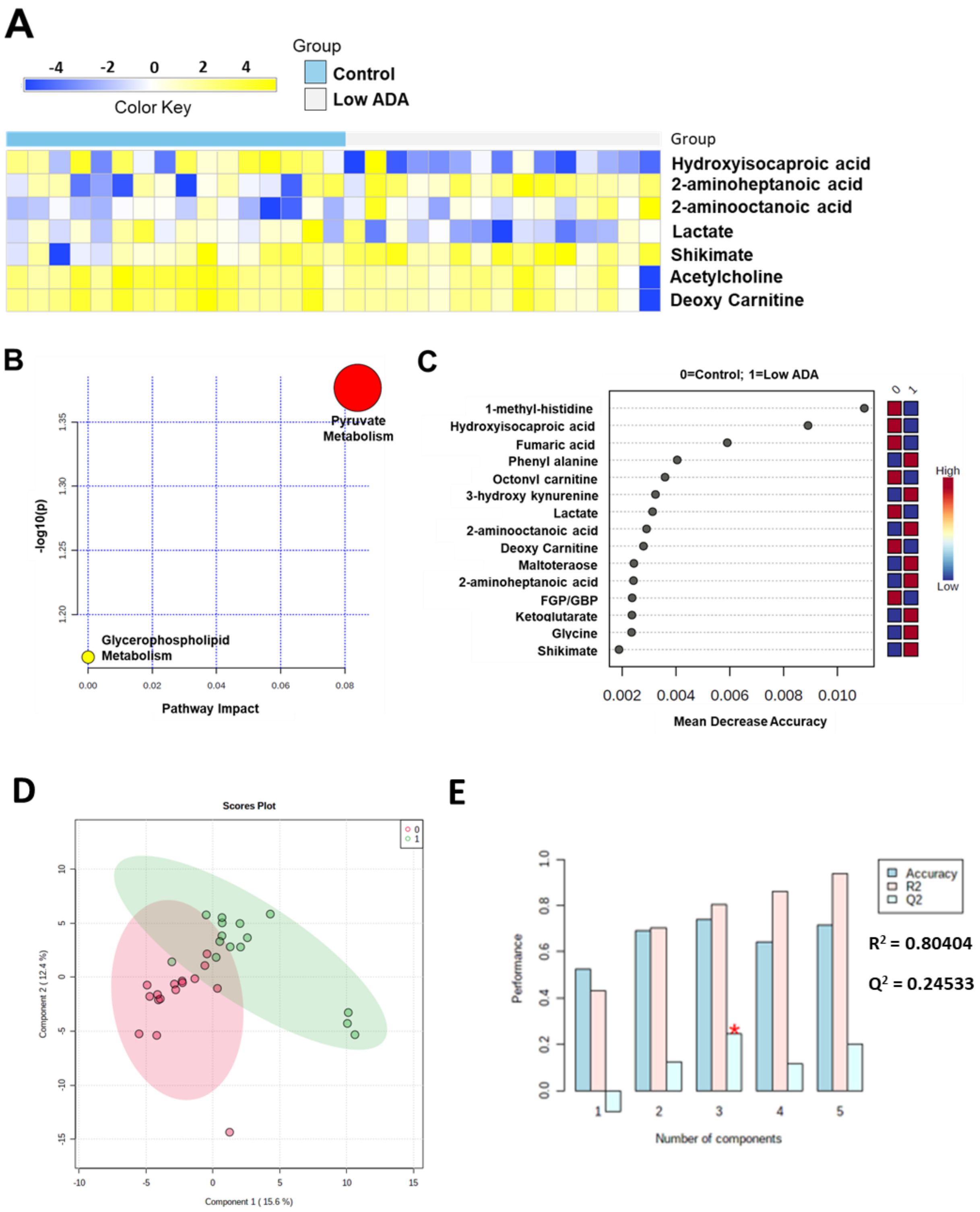

A. Heat map showing the relative levels of 7 significantly altered metabolites at FDR 0.25 with shades of yellow and blue representing the elevated and reduced metabolite levels, respectively (heatmaps were made in Microsoft Excel). **B.** Pathway analysis showing altered metabolic pathways in RA Patients with low ADA levels compared to healthy controls. The size of the circle represents the impact of each pathway and color represents the level of significance (highest in red to lowest in yellow). **C.** Ranking of significant metabolites based on random forest analysis for their ability to stratify RA patients with low ADA levels from healthy controls (ranked by the mean decrease in classication accuracy when they are permuted). **D.** PLS-DA clustering given by score plot (0=Control, 1=Low ADA) and **E.** cross validation of PLS-DA of RA patients with healthy controls and Low ADA levels (red star indicates the best classifier based on accuracy, R^2, and Q^2 values) (Figures B, C, D, and E were generated using the web-based tool Metaboanalyst https://www.metaboanalyst.ca/).

Figure S7a: Targeted Metabolomics data showing altered metabolic signatures in RA patients with high ADA levels compared to healthy controls.

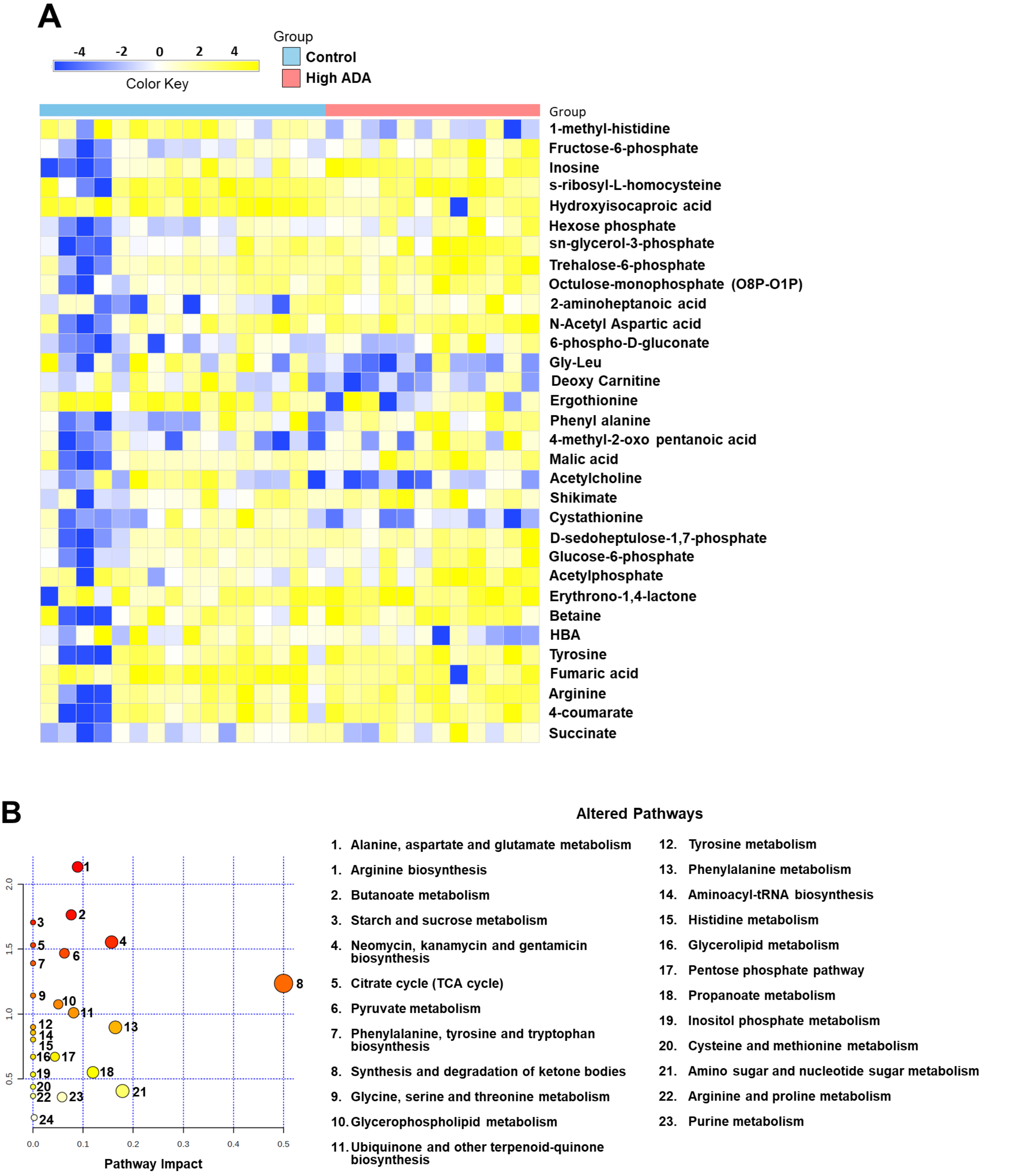

A. Heat map showing the relative levels of 32 significantly altered metabolites at FDR 0.25 with shades of yellow and blue representing the elevated and reduced metabolite levels, respectively (heatmaps were made in Microsoft Excel). **B.** Pathway analysis showing altered metabolic pathways in RA Patients with high ADA levels compared to healthy controls. The size of the circle represents the impact of each pathway and color represents the level of significance (highest in red to lowest in yellow) (Figures B was generated using the web-based tool Metaboanalyst https://www.metaboanalyst.ca/).

Figure S7b: Targeted Metabolomics data showing altered metabolic signatures in RA patients with high ADA levels compared to healthy controls.

C

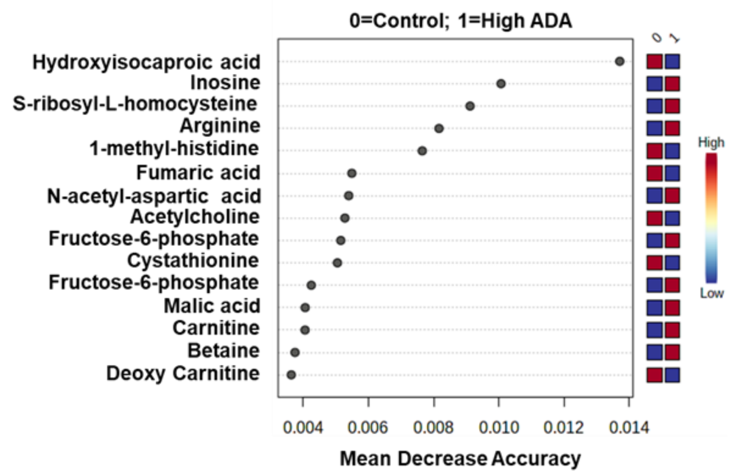

D

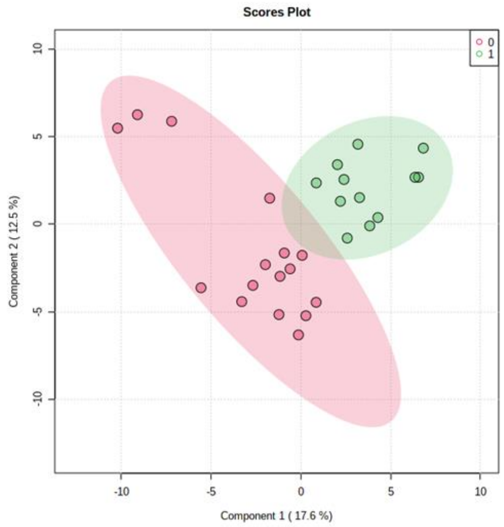

E

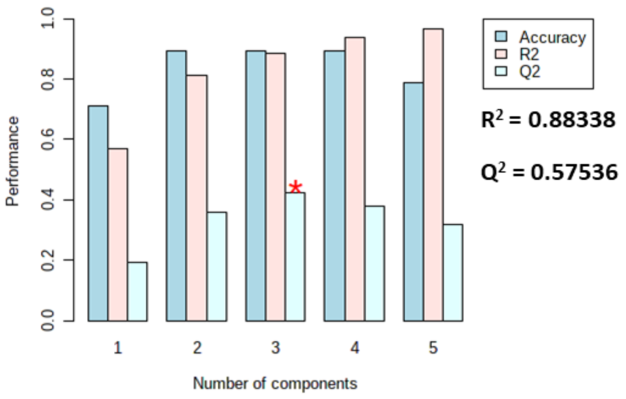

C. Ranking of significant metabolites based on random forest analysis for their ability to stratify RA patients with high ADA levels from healthy controls (ranked by the mean decrease in classication accuracy when they are permuted). **D.** PLS-DA clustering given by score plot (0=Control, 1=High ADA) and **E.** cross validation of PLS-DA of RA patients with healthy controls and High ADA levels (red star indicates the best classifier based on accuracy, R^2, and Q^2 values) (Figures C, D, and E were generated using the web-based tool Metaboanalyst https://www.metaboanalyst.ca/).

Figure S8: Comparative pathway analysis of High ADA and Low ADA metabolomics datasets of current study with High ADA and Low ADA expressing Transcriptomics datasets from GEO database

(Venn Diagrams were created using an online tool https://www.molbiotools.com/listcompare.php).

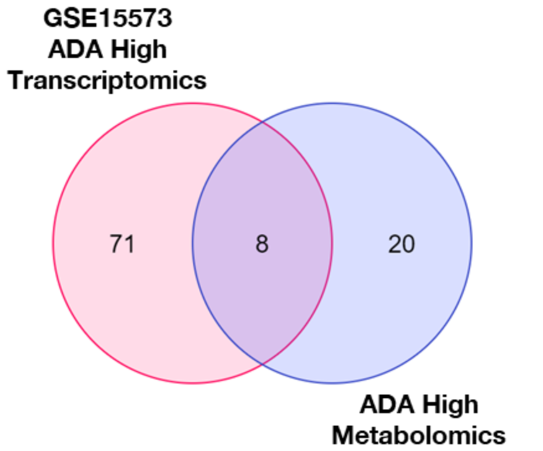

Pyruvate metabolism
Citrate cycle (TCA cycle)
Glycerolipid metabolism
Histidine metabolism
Cysteine and methionine metabolism
Arginine and proline metabolism
Amino sugar and nucleotide sugar metabolism
Aminoacyl-tRNA biosynthesis

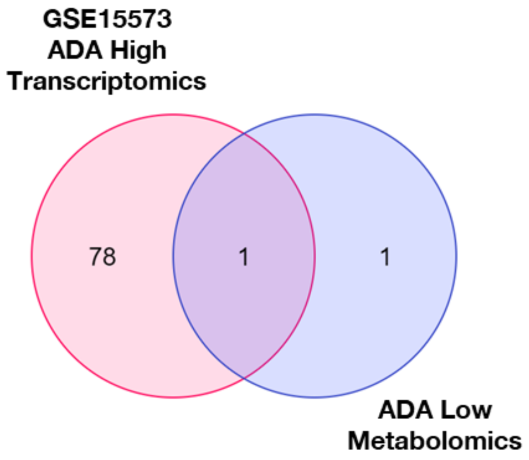

Pyruvate metabolism

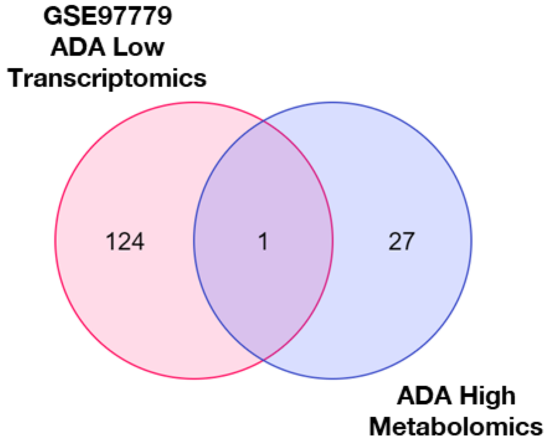

Pyruvate metabolism

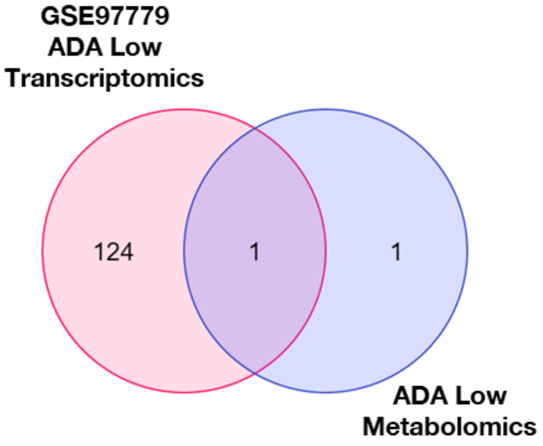

Pyruvate metabolism

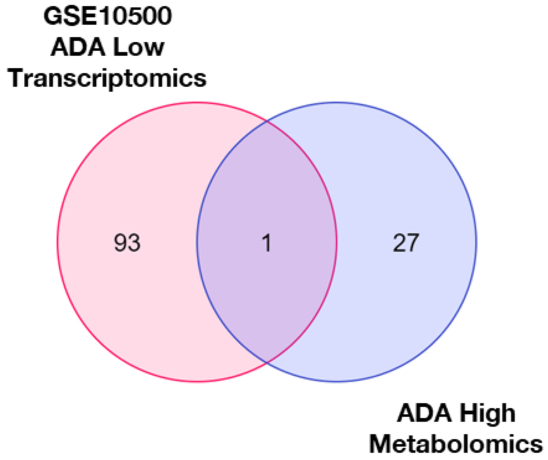

Pentose Phosphate Pathway

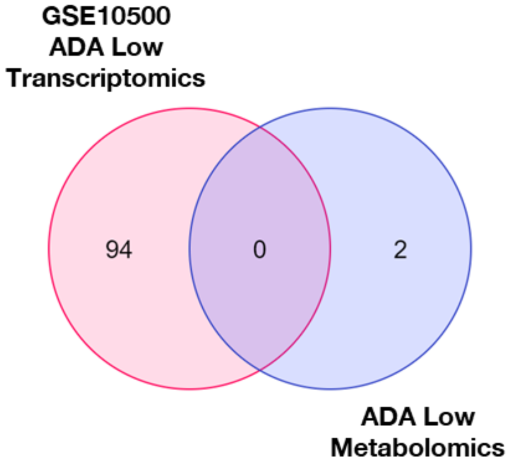

Figure S9: Comparative pathway analysis of serum metabolic datasets of current study with metabolomic datasets of RA patients treated with Rituximab (Biologics) and Methotrexate (DMARD).

PATHWAYS	Control Vs RA	Rituximab Treatment		Methotrexate Treatment
		Before	After	
	Current Study	Shannon R Sweeney etal.		Ryan S. Funk etal
Glycerolipid metabolism	sn-glycerol-3-phosphate ↑	Glycerol ↑	Glycerol ↓	
Glycerophospholipid metabolism	sn-glycerol-3-phosphate ↑			Phosphotidyl inositol 32:0 ↓
Glycolysis/Gluconeogenesis	Lactate ↓	Glucose ↓	Glucose ↓	
Histidine Metabolism	1-methyl Histidine ↓	β-alanine ↓	β-alanine ↑	
Phenylalanine and Tyrosine metabolism	Phenylalanine ↑	Acetoacetate ↓, Phenylalanine ↓	Acetoacetate ↓, Phenylalanine ↓	
Phospholipid Biosynthesis	Acetylcholine ↓	Choline ↓	Choline ↓	
Purine Metabolism	Inosine ↑	Glycine ↓, Hypoxanthine ↓, Xanthine ↑	Glycine, Hypoxanthine ↓, Xanthine ↓	Inosine monophosphate ↑
Pyruvate metabolism	Lactate ↓	Pyruvate ↑	Pyruvate ↑	

PATHWAYS	Control Vs Low ADA	Rituximab Treatment		Methotrexate Treatment
		Before	After	
	Current Study	Shannon R Sweeney etal.		Ryan S. Funk etal
Phospholipid Biosynthesis	Acetylcholine ↓	Choline ↓	Choline ↓	
Glycolysis/Gluconeogenesis	Lactate ↓	Glucose ↓	Glucose ↓	
Pyruvate metabolism	Lactate ↓	Pyruvate ↑	Pyruvate ↑	

PATHWAYS	Control Vs High ADA	Rituximab Treatment		Methotrexate Treatment
		Before	After	
	Current Study	Shannon R Sweeney etal.		Ryan S. Funk etal
Arginine biosynthesis	Arginine ↑	Glutamine ↑, Ornithine ↓	Glutamine ↓, Ornithine ↓	
Arginine and Proline Metabolism	Succinate ↑	Glycine ↓, Urea ↓	Glycine ↓, Urea ↑	
Aspartate metabolism	Arginine ↑, Fumaric Acid ↓, N-acetyl Aspartic Acid ↑	Aspargine ↓, Aspartate ↑, β-alanine ↓	Aspargine ↓, Aspartate ↑, β-alanine ↑	Inosine monophosphate ↑
Betaine Metabolism	Betaine ↑	Betaine ↓	Betaine ↓	
Carnitine synthesis	Succinate ↑	Carnitine ↓, Lysine ↓	Carnitine ↓, Lysine ↓	
Citric acid cycle	Malic Acid ↑	Pyruvate ↑	Pyruvate ↑	
Fatty Acid Biosynthesis	Hydroxy Butyric Acid ↓	3-Hydroxybutyrate ↑	3-Hydroxybutyrate ↓	
Galactose metabolism	Glucose-6-Phosphate↑	Glycerol ↓	Glycerol ↓	Fructose ↑
Gluconeogenesis	Glucose-6-Phosphate ↑	Pyruvate ↑, Glucose ↓	Pyruvate ↑, Glucose ↓	
Glutamate Metabolism	Succinate ↑, Fructose-6-Phosphate ↑	Glutamate ↑, Glutamine ↑, Pyruvate ↑	Glutamate ↓, Glutamine ↓, Pyruvate ↑	
Glycerolipid metabolism	sn-glycerol-3-phosphate ↑	Glycerol ↑	Glycerol ↓	
Glycerophospholipid metabolism	sn-glycerol-3-phosphate ↑			Phosphotidyl inositol 32:0 ↓
Histidine Metabolism	1-methyl Histidine, Ergothionine ↓	β-alanine ↓	β-alanine ↑	
Homocysteine Degradation	Cystathionine ↓	Serine ↓	Serine ↓	
Methionine Metabolism	Betaine ↑, Cystathionine ↓	Betaine ↓, Choline ↓	Betaine ↓, Choline ↓	
Oxidation of branched chain fatty acids	Succinate ↑	O-Acetyl carnitine ↓	O-Acetyl carnitine ↓	
Phenylalanine and Tyrosine metabolism	Tyrosine ↑, Phenylalanine ↑	Acetoacetate ↓, Phenylalanine ↓	Acetoacetate ↓, Phenylalanine ↓	
Phospholipid Biosynthesis	Acetylcholine ↓	Choline ↓	Choline ↓	
Pyruvate metabolism	Acetylphosphate ↑, Malic Acid ↑	Pyruvate ↑	Pyruvate ↑	
Sphingolipid metabolism		Serine ↓	Serine ↓	
Starch and Sucrose Metabolism	Glucose-6-Phosphate ↑			Fructose ↑
Valine, leucine and isoleucine biosynthesis		Isoleucine ↓	Isoleucine ↑	
Valine, leucine, and Isoleucine degradation	Succinate ↑, 4-Methyl-2-oxo pentanoic acid ↑	Methylmalonate ↑, Oxoisocaproate ↓	Methylmalonate ↑, Oxoisocaproate ↓	

Table S1: Altered significant serum metabolites between RA patients and healthy controls with increasing FDR (at FDR 0.25).

Metabolite	HMDB ID	KEGG ID	Mode	MRM		p Value	FDR	AUC Score
				Precursor ion	Product ion			
2-Aminoheptanoicacid	NA	NA	Positive	146.1176	100.1	0.00024272	0.030097	0.778
Hydroxyisocaproic acid	HMDB0000746	NA	Negative	131.006	85.1	0.00054523	0.033804	0.856
shikimate	HMDB0003070	C00493	Negative	173	93	0.0023987	0.078861	0.759
S-ribosyl-L-homocysteine	METPA0405	C03539	Positive and Negative	268.0849	88	0.0025439	0.078861	0.759
1-Methyl-Histidine	HMDB0000001	C01152	Positive	170.0924	124	0.0053824	0.1116	0.817
Acetylcholine	HMDB0000895	C01996	Positive	146.1176	43.02	0.0071111	0.1116	0.745
Deoxy carnitine	NA	NA	Positive	146.1176	60.1	0.0075035	0.1116	0.787
Cystathionine	HMDB0000099	C02291	Positive	223.0747	134.1	0.007844	0.1116	0.722
2-Aminooctanoicacid	NA	NA	Positive	160.1332	55.3	0.008432	0.1116	0.748
Phenyl alanine	NA	NA	Positive	166.0963	103.1	0.0098957	0.1116	0.727
Inosine	HMDB0000195	C00294	Negative	267	135	0.0098998	0.1116	0.773
sn-glycerol-3-phosphate	HMDB0000126	C00093	Negative	171	79	0.01633	0.16874	0.718
Erythrono-1,4-lactone	HMDB0000349	NA	Positive	119.11	91.1	0.019919	0.19	0.681
Lactate	HMDB0000190	C00256	Negative	89	43.2	0.022356	0.19801	0.734
2-Hydroxypyridine	HMDB0013751	C02502	Positive	96.0444	51.1	0.029303	0.24224	0.676

Table S2: Altered significant serum metabolites between RA patients with Low ADA and High ADA activity and healthy controls with increasing FDR (at FDR 0.25).

Metabolite	HMDB ID	KEGG ID	Mode	MRM		p Value	FDR
				Precursor ion	Product ion		
2-Aminoheptanoicacid	NA	NA	Positive	146.12	100.10	0.0012	0.0803
Hydroxyisocaproic acid	HMDB0000746	NA	Negative	131.01	85.10	0.0013	0.0803
Fructose-6-Phosphate	HMDB0000124	C00085	Negative	259.00	97.10	0.0020	0.0812
Hexose-phosphate	NA	NA	Negative	259.00	79.00	0.0028	0.0878
S-ribosyl-L-homocysteine	METPA0405	C03539	Positive	268.08	88.00	0.0043	0.1065
S-ribosyl-L-homocysteine	METPA0405	C03539	Negative	266.00	134.00	0.0043	0.1065
shikimate	HMDB0003070	C00493	Negative	173.00	93.00	0.0106	0.1929
Inosine	HMDB0000195	C00294	Negative	267.00	135.00	0.0109	0.1929
Acetylcholine	HMDB0000895	C01996	Positive	146.12	43.02	0.0155	0.2395
Deoxy carnitine	NA	NA	Positive	146.12	60.10	0.0192	0.2477
1-Methyl-Histidine	HMDB0000001	C01152	Positive	170.09	124.00	0.0200	0.2477

Table S3: List of all Significant Pathways from the pathway analysis of the all three published metabolomic datasets

	Study	Metabolic Pathways	P value
1	**LC-MS-based serum metabolomics reveals a distinctive signature in patients with rheumatoid arthritis** by Li et al.	Aminoacyl-tRNA biosynthesis	9.28E-06
		Arginine biosynthesis	9.29E-05
		Alanine, aspartate and glutamate metabolism	0.000792
		Porphyrin and chlorophyll metabolism	0.017876
		Arginine and proline metabolism	0.028007
		Phenylalanine, tyrosine and tryptophan biosynthesis	0.028113
		Nitrogen metabolism	0.041899
		D-Glutamine and D-glutamate metabolism	0.041899
2	**Exploration of the serum metabolite signature in patients with rheumatoid arthritis using gas chromatography–mass spectrometry** by Zhou et al.	Aminoacyl-tRNA biosynthesis	1.68E-11
		Valine, leucine and isoleucine biosynthesis	1.05E-09
		Biosynthesis of unsaturated fatty acids	0.000558
		Valine, leucine and isoleucine degradation	0.00092
		Phenylalanine, tyrosine and tryptophan biosynthesis	0.002266
		Phenylalanine metabolism	0.01577
		Cysteine and methionine metabolism	0.026344
3	**Diagnostic properties of metabolic perturbations in rheumatoid arthritis** by Madsen et al.	Aminoacyl-tRNA biosynthesis	5.19E-05
		Glycine, serine and threonine metabolism	0.011364
		Valine, leucine and isoleucine biosynthesis	0.040642

Table S4: Altered serum metabolites between RA patients with Low ADA and High ADA activity (at FDR 0.25).

Metabolite	HMDB ID	KEGG ID	Mode	MRM		p Value	FDR	AUC Score
				Precursor ion	Product ion			
Fructose-6-phosphate	HMDB0000124	C00085	Negative	259	97.1	0.0037946	0.2385	**0.856**
Hexose-phosphate	HMDB0001401	C00092	Negative	259	79	0.0038468	0.2385	**0.828**

Table S5: Altered serum metabolites between RA patients with Low ADA activity and healthy controls with increasing FDR (at FDR 0.25).

Metabolite	HMDB ID	KEGG ID	Mode	MRM		p Value	FDR	AUC Score
				Precursor ion	Product ion			
Hydroxyisocaproic acid	HMDB0000746	NA	Negative	131.006	85.1	0.0005	0.062	0.829
2-Aminoheptanoicacid	NA	NA	Positive	146.1176	100.1	0.0017	0.108	0.808
2-Aminooctanoicacid	NA	NA	Positive	160.1332	55.3	0.0078	0.230	0.767
Lactate	HMDB0000190	C00256	Negative	89	43.2	0.0081	0.230	0.779
shikimate	HMDB0003070	C00493	Negative	173	93	0.0111	0.230	0.763
Acetylcholine	HMDB0000895	C01996	Positive	146.1176	43.02	0.0118	0.230	0.771
Deoxy carnitine	NA	NA	Positive	146.1176	60.1	0.0130	0.230	0.796

Table S6: Altered serum metabolites between RA patients with High ADA activity and healthy controls with increasing FDR (at FDR 0.25).

Metabolite	HMDB ID	KEGG ID	Mode	MRM		p Value	FDR	AUC Score
				Precursor	Product ion			
1-Methyl-Histidine	HMDB0000001	C01152	Positive	170.1	124	0.000628	0.05	0.844
Fructose-6-Phosphate	HMDB0000124	C00085	Negative	259	97.1	0.001128	0.05	0.849
Inosine	HMDB0000195	C00294	Negative	267	135	0.001787	0.05	0.865
S-ribosyl-L-homocysteine	METPA0405	C03539	Positive	268.1	88	0.001884	0.05	0.844
S-ribosyl-L-homocysteine	METPA0405	C03539	Negative	266	134	0.001884	0.05	0.844
Hydroxyisocaproic acid	HMDB0000746	NA	Negative	131	85.1	0.002357	0.05	0.891
Hexose-phosphate	NA	NA	Negative	259	79	0.002601	0.05	0.818
sn-glycerol-3-phosphate	HMDB0000126	C00093	Negative	171	79	0.007594	0.13	0.786
Trehalose-6-Phosphate	HMDB0001124	C00689	Negative	421	79	0.008492	0.13	0.823
octulose-monophosphate O8P-O1P	NA	NA	Negative	319	97	0.009833	0.14	0.797
2-Aminoheptanoicacid	NA	NA	Positive	146.1	100.1	0.011205	0.14	0.740
N-Aetyl Aspartic acid	HMDB0000812	C01042	Negative	174	88.1	0.013835	0.14	0.781
6-phospho-D-gluconate	HMDB0001316	C00345	Negative	275	97	0.017312	0.14	0.740
Gly-Leu	HMDB0000759	C02155	Positive	189.1	171	0.017416	0.14	0.771
Deoxy carnitine	NA	NA	Positive	146.1	60.1	0.017518	0.14	0.776
Ergothionine	HMDB0003045	C05570	Positive	230.1	186.1059	0.017733	0.14	0.734
Phenyl alanine	NA	NA	Positive	166.1	103.1	0.018122	0.14	0.755
4-Methyl-2-oxo pentanoic acid	NA	NA	Positive	131.1	43.06	0.019597	0.14	0.755
Malic acid	HMDB0000744	C03668	Negative	133	115.1	0.021009	0.14	0.729
Acetylcholine	HMDB0000895	C01996	Positive	146.1	43.02	0.022195	0.14	0.714
shikimate	HMDB0003070	C00493	Negative	173	93	0.023069	0.14	0.755
Cystathionine	HMDB0000099	C02291	Positive	223.1	134.1	0.024056	0.14	0.745
D-sedoheptulose-1-7-phosphate	NA	NA	Negative	289	97	0.024315	0.14	0.760
Glucose-6-Phosphate	HMDB0001401	C00092	Negative	259	97	0.026942	0.14	0.703
Acetylphosphate	HMDB0001494	C00227	Negative	139	79	0.028126	0.14	0.724
Erythrono-1,4-lactone	HMDB0000349	NA	Positive	119.1	91.1	0.028416	0.14	0.750
Betaine	HMDB0000043	C00719	Positive	118.1	59.2	0.037563	0.17	0.724
HBA	HMDB0000357	C01089	Negative	103	59.1	0.038502	0.17	0.667
Tyrosine	HMDB0000158	C00082	Positive	182.1	91	0.038726	0.17	0.688
Fumaric acid	HMDB0000134	C00122	Negative	115	71.1	0.040439	0.17	0.734
Arginine	HMDB0000517	C00062	Positive	175.1	60.2	0.046664	0.19	0.745
4-Coumarate	HMDB0002035	C00811	Positive	165	119	0.055076	0.22	0.693
succinate	HMDB0000254	C00042	Negative	117	73	0.060176	0.23	0.708
